# Towards direct detection of tetracycline residues in milk with a gold nanostructured electrode

**DOI:** 10.1371/journal.pone.0287824

**Published:** 2023-06-27

**Authors:** Magdalena R. Raykova, Katie McGuire, William J. Peveler, Damion K. Corrigan, Fiona L. Henriquez, Andrew C. Ward

**Affiliations:** 1 Civil and Environmental Engineering, University of Strathclyde, Glasgow, United Kingdom; 2 School of Chemistry, University of Glasgow, Glasgow, United Kingdom; 3 Pure and Applied Chemistry, University of Strathclyde, Glasgow, United Kingdom; 4 School of Health and Life Sciences, University of the West of Scotland, Paisley, United Kingdom; Hamadan University of Medical Sciences, ISLAMIC REPUBLIC OF IRAN

## Abstract

Tetracycline antibiotics are used extensively in veterinary medicine, but the majority of the administrated dose is eliminated unmodified from the animal through various excretion routes including urine, faeces and milk. In dairy animals, limits on residues secreted in milk are strictly controlled by legislation. Tetracyclines (TCs) have metal chelation properties and form strong complexes with iron ions under acidic conditions. In this study, we exploit this property as a strategy for low cost, rapid electrochemical detection of TC residues. TC-Fe(III) complexes in a ratio of 2:1 were created in acidic conditions (pH 2.0) and electrochemically measured on plasma-treated gold electrodes modified with electrodeposited gold nanostructures. DPV measurements showed a reduction peak for the TC-Fe(III) complex that was observed at 50 mV (vs. Ag/AgCl QRE). The limit of detection in buffer media was calculated to be 345 nM and was responsive to increasing TC concentrations up to 2 mM, added to 1 mM FeCl_3_. Whole milk samples were processed to remove proteins and then spiked with tetracycline and Fe(III) to explore the specificity and sensitivity in a complex matrix with minimal sample preparation, under these conditions the LoD was 931 nM. These results demonstrate a route towards an easy-to-use sensor system for identification of TC in milk samples taking advantage of the metal chelating properties of this antibiotic class.

## Introduction

Maximum drug residue limits in food (MRLs) are strictly controlled by legislation in the majority of countries and territories [1]. The responsibility for ensuring that food products are safe for consumers lies with the producers and processors. To address this, several commercially available techniques exist to screen for drug residues and ensure quality control of food products, including laboratory services and on site tests to ensure confidence of supply [2–4]. A key drawback of existing technologies lies in their relatively high cost, compared to the value of the final product [5]. Furthermore, many tests have a time-to-result of several hours, which can add problematic delay into fresh food production cycles [6]. In dairy farms, contamination of the farm bulk tank with milk containing residues, such as tetracyclines, presents a threat to confidence of supply, results in financial losses to the farmer and dairy industry and disposal of the contaminated product imposes a risk to the environment [7].

Tetracyclines (TCs) are broad-spectrum antibiotics widely used in human and veterinary medicine that are active against a range of Gram-positive and Gram-negative bacteria [8, 9]. The TC group of drugs contain: tetracycline (TC), oxytetracycline (OTC), chlortetracycline (CTC), and doxycycline (DXY), and are predominantly used to treat dermatological conditions, mastitis and respiratory diseases caused by *Pasteurella multocida*, *Mannheimia haemolytica* or *Histophilus somni* [10]. Tetracycline drugs are not strongly metabolised when administrated, therefore high drug residues are found in faeces, urine and milk of treated animals [1, 11, 12]. OTC, CTC and DXY are excreted in their unchanged active form, whereas 5% of TC is metabolised to 4-epitetracycline prior to excretion, which is less active that the parent drug [13]. All drugs within the tetracycline class have a milk MRL of 100 μg/L (which equates to 225 nM for TC) and excretion of unmetabolized TC residues requires that animals for food consumption must undergo a withdrawal period of several days after treatment to ensure the food product does not contain residue concentrations above the MRL [14, 15].

TC residues also pose potential problems in wastewater effluents. A number of reports disclose that large concentrations of the parent compounds are found in wastewater, as well as in the environment as a consequence from using manure as fertiliser or disposal of contaminated milk from dairy farms, due to wastewater treatment plants (WWTPs) being inefficient at removing such pollutants [5, 16–19]. For instance, TC and OTC in effluent, after four-stage treatment in a WWTP have been found at 25.8 ng/L and 64.5 ng/L, respectively [20]. More concentrated residues have also been found in hospital and drug manufacturing effluents, ranging between 50 μg/L and 270 μg/L [21]. Reported concentrations of tetracycline in liquid manure are as high as 20 mg/L [22]. For more details, readers are referred to a recent and comprehensive review of TCs fate and occurrence in environmental samples including details on samples type and country of origin by Scaria et al. [23].

Iron is an abundant transition metal widely present in the environment, typically in the μM range [24]. There are two oxidation states of the metal, Fe(II) and Fe(III), both are stable in various environmental conditions with Fe(III) prevailing in higher oxygen content media where organic compounds are available for chelation [25, 26]. In nature, iron exists in the form of ore minerals or dissolved ions in water [27, 28]. Traces vary with pH of aquatic environments, with elevated concentrations found in acidic aqueous conditions [29]. In analytical electrochemistry, Fe(III) ions are widely exploited in redox couples to probe electrode-electrolyte reactions. For example, a common approach involves the potassium ferricyanide/ferrocyanide [Fe(CN)_6_^3-^]/[Fe(CN)_6_^4-^] couple to measure changes in charge at an electrode surface following binding or immobilisation of an analyte [30, 31]. The use of FeCl_3_ in a solution can also produce free ferric ions that result in redox peaks that could also be observed on different types of electrodes [32, 33].

A unique property of TC drugs is their ability to rapidly form complexes with transition metal ions, such as Fe(III), in solutions [16, 34–36]. Wang et al. explored the interactions between tetracyclines and Fe(III) ions and reported that Fe(III) is bound by the antibiotics (at 1:1 or 2:1 ratio, respectively) at pH > 7.0 and promotes oxidation of these drugs over time [37, 38]. This is an important role of the metal in the environment, however, Jačić et al., state that at pH < 5 the Fe(III)-TC complex at 1:1 ratio is very stable and TC is not prone to oxidation, hence more persistent [39]. All studies suggest that there are two sites for metal binding: the dimethylamine group via one of the four rings of the TC’s naphthalene core or via the 1,3-diketone groupings branching out from the rings [35, 40]. Binding of different metal ions is dependent upon pH and charge of the ion. The extent to which compounds bind metal ions and form a complex can be expressed by the formation constant logK [41]. According to sources from literature, TC binds with affinity towards Fe(III) logK value of 6.8 to 9.9 and 13.4, respectively [42–44]. Ethylenediamine tetra acetic acid (EDTA), commonly used as a metal chelating agent for heavy metal poising and various pharmaceutical applications, has six potential sites in its chemical structure for metal chelation [45, 46]. In contrast to TC, EDTA binds to Fe(III) with logK of 25 and chelates to metal ions in a ratio 1:1 [41, 47]. This higher the complex formation constant, the tougher the complex would split into its component ions. EDTA is also used in milk sample processing prior to analysis for antibiotics, tetracyclines specifically, to break the bonds of the known complexes that TC develops with Mg^2+^ and Ca^2+^ ions [48]. Hence, the strong binding affinity of EDTA provides a benchmark against which to compare TC-Fe binding process. In addition, if milk sample preparation includes a step of EDTA addition when concentrating TC analyte, residues of the metal chelator may remain in the samples and interfere with further iron complexation initiation with TC. Therefore, it is essential to examine the behaviour of EDTA in the presence of TC-Fe(III) complex in a solution to explore a potential competition in an assay.

**Fig 1:**
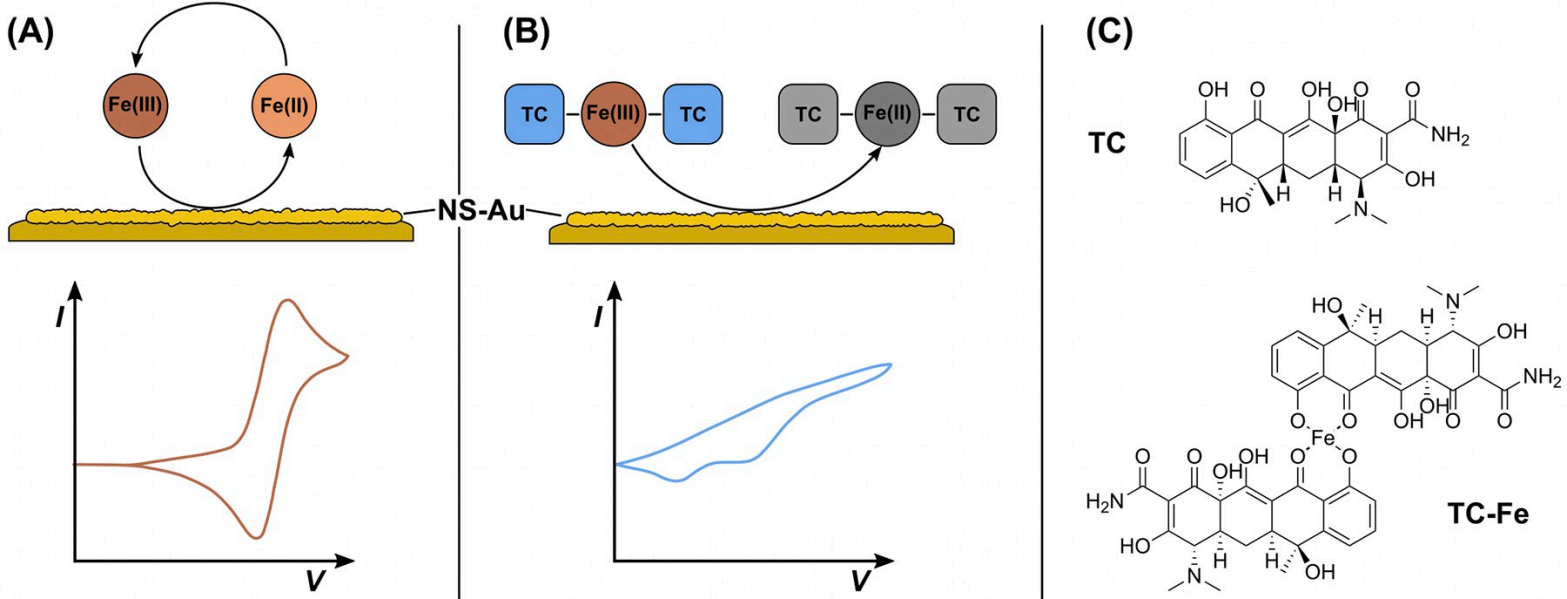
Direct electrochemical detection schema for Fe-TC complexes and nanostructured gold electrode surfaces (AuNS). (A) Fe(III)/Fe(II) reversible redox reaction occurs at Nanostructured Gold (AuNS) electrodes. (B) TC forms a complex with Fe(III), resulting in irreversible reduction reactions at the NS-Au surface. (C) Structure of TC molecule and putative structure of TC-Fe complex.

In this study, we explore a direct electrochemical detection technique for the TC-Fe(III) complex using cyclic voltammetry (CV) and differential pulse voltammetry (DPV) directly on gold (Au) electrodes coated with gold nanostructures (AuNS) (Fig 1). Electrochemical detection directly into a solution is possible if the compound in question is electrochemically active on its own or when combined with another substance. Using this approach whole pasteurised milk samples were also analysed as an exemplar towards use by food producers. The analysis of TC-Fe(III) has been reported in the literature using methods such as UV-Vis or chromatography with most papers describing this complexation being more than 10 years old, however, key to our study. We, therefore, propose an alternative analytical approach for the analysis of the complex electrochemically on a gold electrode surface which gold nanostructures were incorporated on. This electrochemical method proved to be rapid (< 5 min) with no requirement for incubation time on the electrode. It also relies on the abundant and distinguishable electrochemical profile of TC-Fe(III) complex in small acidic samples (500 μL).

## Materials and methods

### Reagents and instruments

Gold (III) chloride hydrate (HAuCl_4_) was purchased from Sigma Aldrich and was used for gold nanoparticles synthesis. FeCl_3_ (used as the Fe(III) ions source), tetracycline (98–102% HPLC), oxytetracycline hydrochloride (OTC), chloramphenicol (CA), penicillin G potassium salt (Pen G), erythromycin (98%) (ERY) and magnesium chloride hexahydrate (MgCl_2_) were purchased from Sigma Aldrich. Ampicillin sodium salt (AMP) was purchased from Applichem. D(+) glucose monohydrate (Glu) and calcium chloride dihydrate (CaCl_2_) were purchased from Scientific laboratory supplies and VWR chemicals, respectively. Ethylenediaminetetraacetic acid (EDTA) was purchased from Acros Organics. Gold electrodes (1 mm in diameter) in the form of an array of 8 electrodes on a polypropylene chip with a common Au counter electrode and a common Ag/AgCl quasi reference electrode (QRE) were obtained to a custom design from FlexMedical Solutions Ltd (Livingston, UK). A PalmSens4 potentiostat was connected to a PalmSens MUX8-R2 multiplexer and used to take measurements of the 8 consecutive channels. Solutions were prepared using ultrapure deionised water (*d*H_2_O) from a Triplered 18.2 MΩ-cm system. Whole fat pasteurised milk was purchased from a local supermarket.

### Gold nanostructures synthesis and imaging

Gold (Au) electrodes were plasma-treated using a Diener Zepto plasma-surface machine for 62 secs at 0.8 mbar pressure for 1 cycle at 50% power, prior any modification or analysis. was used for gold AuNS synthesis was carried out following the method described by Wan et al. [49]. Briefly, the gold electrode surface was activated in 0.5 M H_2_SO_4_ using CV and then AuNS were electrodeposited in 5 mM HAuCl_4_ solution prepared in 0.5 M H_2_SO_4_ using chronoamperometry (CA) at -0.5 V for 120s. Images of the working electrode before and after AuNS electrodeposition were obtained using Philips/FEI XL30 environmental scanning electron microscope (ESEM) operating at 20kV in secondary electron detection mode.

### TC-Fe(III) complex synthesis and analysis

#### Electrochemical analysis

Stock solutions (10 mM) of FeCl_3_, TC and EDTA were prepared in *d*H_2_O adjusted to pH 2.0 with 1 M HCl. Using the stock solutions, a range of TC concentrations were added to 10 mM KCl containing 1 mM iron ions and solutions were scanned on the prepared electrode surfaces (*n* = 8) in an increasing TC concentration order. All electrochemical measurements in this work were performed using CV at scan rate 10 mV/s in the range between -0.4 V and 0.6 V starting at -0.4 V and initially sweeping towards more positive potentials. DPV was also applied at a scan rate of 50 mV/s with pulse width of 50 ms, and pulse amplitude of 50 mV in a 0.6 V to -0.2 V potential window. Solutions were measured electrochemically immediately after preparation.

#### UV-Vis analysis

The absorbance spectra of FeCl_3_ and TC separately and in a combination were obtained using a UV-Vis spectrometer HACH LANGE DR 6000 in solution media adjusted to pH 2 with 1 M HCl. The absorbance of each solution was measured at 444 nm.

### Milk samples analysis

Commercial whole milk was purchased from the local supermarket. A volume of 1.5 mL milk was mixed with 500 μL of 1 M HCl and shaken by hand, causing an immediate protein precipitation. The sample was centrifuged for 15 minutes at 7000 rpm, and the supernatant was then filtered through a 0.45 μm syringe filter. Finally, 100 μL of the filtered supernatant were diluted to 10 mL (1:100 dilution) using the pH 2.0 adjusted solution. This matrix was then spiked with TC at a range of concentrations of in a set of separate and each solution respectively with FeCl_3_ at concentration of 1 mM. A blank sample was also prepared, with 1 mM FeCl_3_ and with no TC added.

## Results and discussion

### Gold surface baseline characterisation

**Fig 2:**
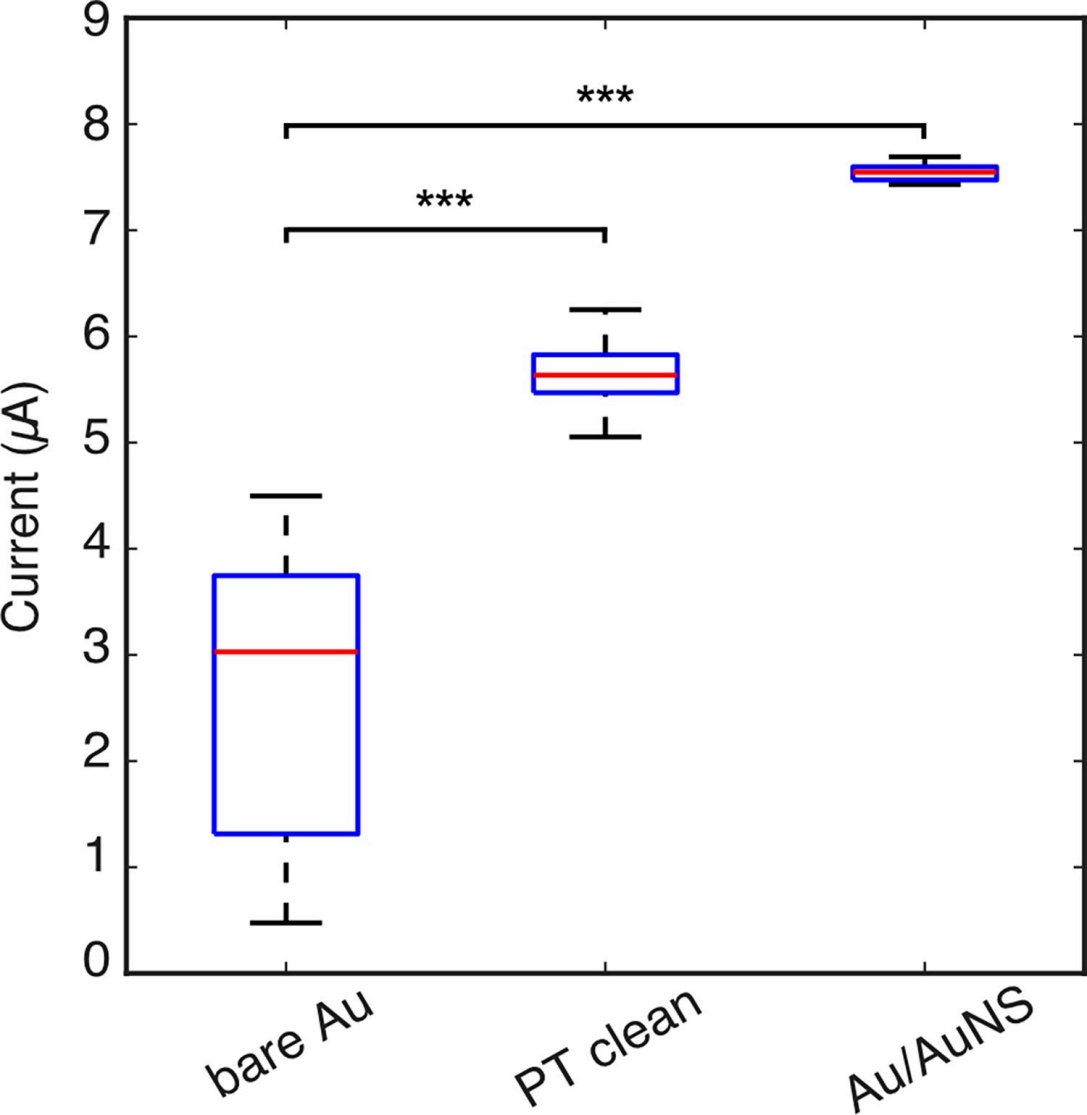
Absolute current measured in 1mM Fe(CN)63-/Fe(CN)64- with 100 mM KCl (*n =* 16, ***P < 0.001, box represents the interquartile range, whiskers represent the range of minimum and maximum measured values, red line is the mean current measured and a single outlier from the Au/AuNS box is shown as red +).

To improve performance, the electrodes were cleaned prior to test measurements with different TC concentrations. Oxygen plasma treatment was found to clean the gold surface better than electrochemical cleaning. A number of methods for deposition of gold nanostructures were tested including physical adsorption, cyclic voltammetry (CV) and chronoamperometry (CA) [50]. The three methods were compared by carrying out electrochemical measurements in 1mM Fe(CN)_6_^3-^/Fe(CN)_6_^4-^ with 100 mM KCl. The synthesis of AuNS directly onto the electrode via electrodeposition using CA combined with plasma treatment significantly improved consistency between electrodes further and enlarge the surface area (Fig 2). This shows an increase in the faradaic current as a result of the highly oxidative cleaning process from the plasma removing contaminants from the gold electrode surface and a further increase in the electrode surface area as a result of the AuNS deposition. The current was significantly higher on AuNS electrodes, contrasted to the same electrodes after plasma treating (P < 0.001, Student’s paired t-test).

**Fig 3:**
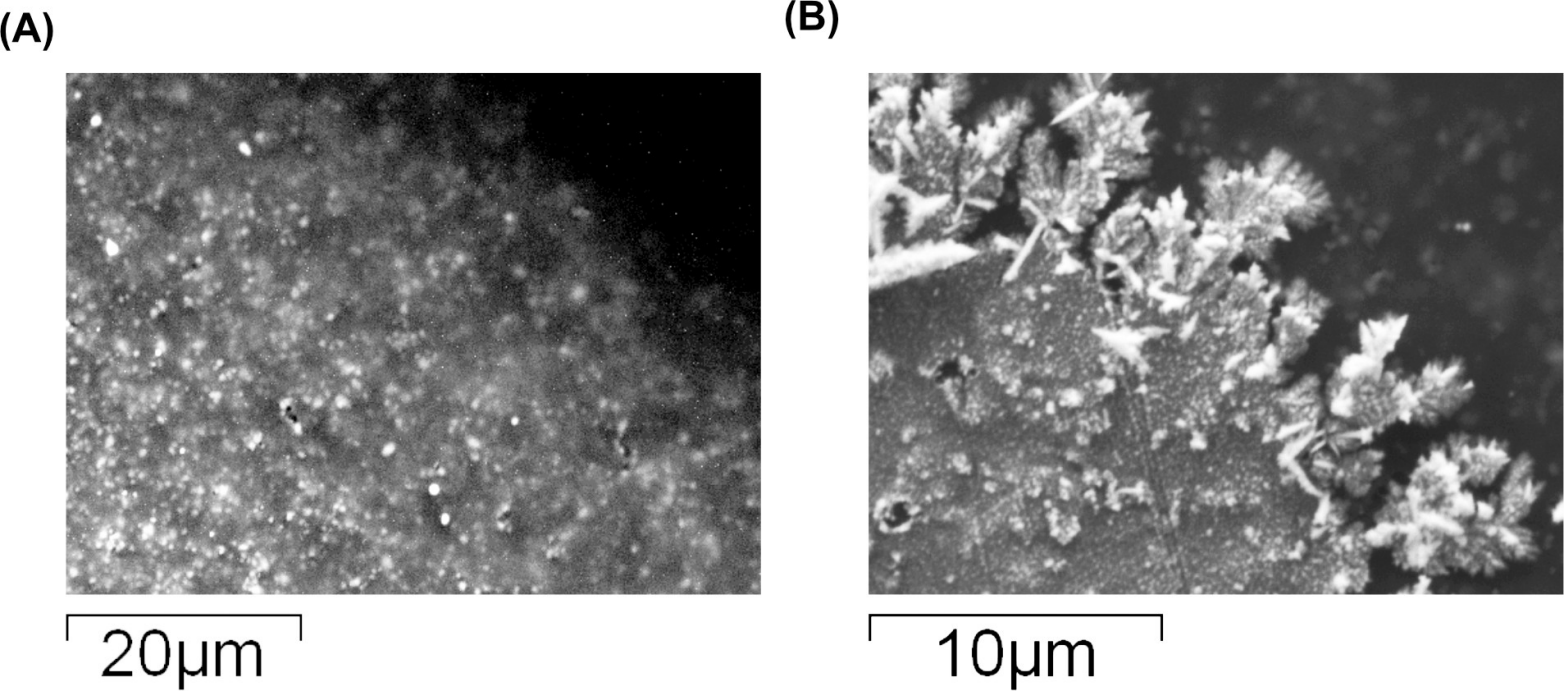
SEM images, scale indicated on image, where (A) edge of bare gold electrode at magnification 2000x; (B) edge of electrode after electrodeposition of AuNS in 5mM HAuCl_4_ solution prepared in 0.5M H_2_SO_4_ using chronoamperometry at magnification 5000x.

The successful synthesis of AuNS on the bare gold electrode surface using CA was also confirmed by SEM imaging. As seen in Fig 3 below, gold nanostructures are more distinguishable at the edge of the working electrode where flower-like or crystal-like structures are observed. These structures are clearly absent in the image containing the edge of the bare gold electrode. An alternative magnification with scales indicated on images can be seen in S1 Fig.

### Electrochemical characterisation of TC and Fe(III) complexes

**Fig 4:**
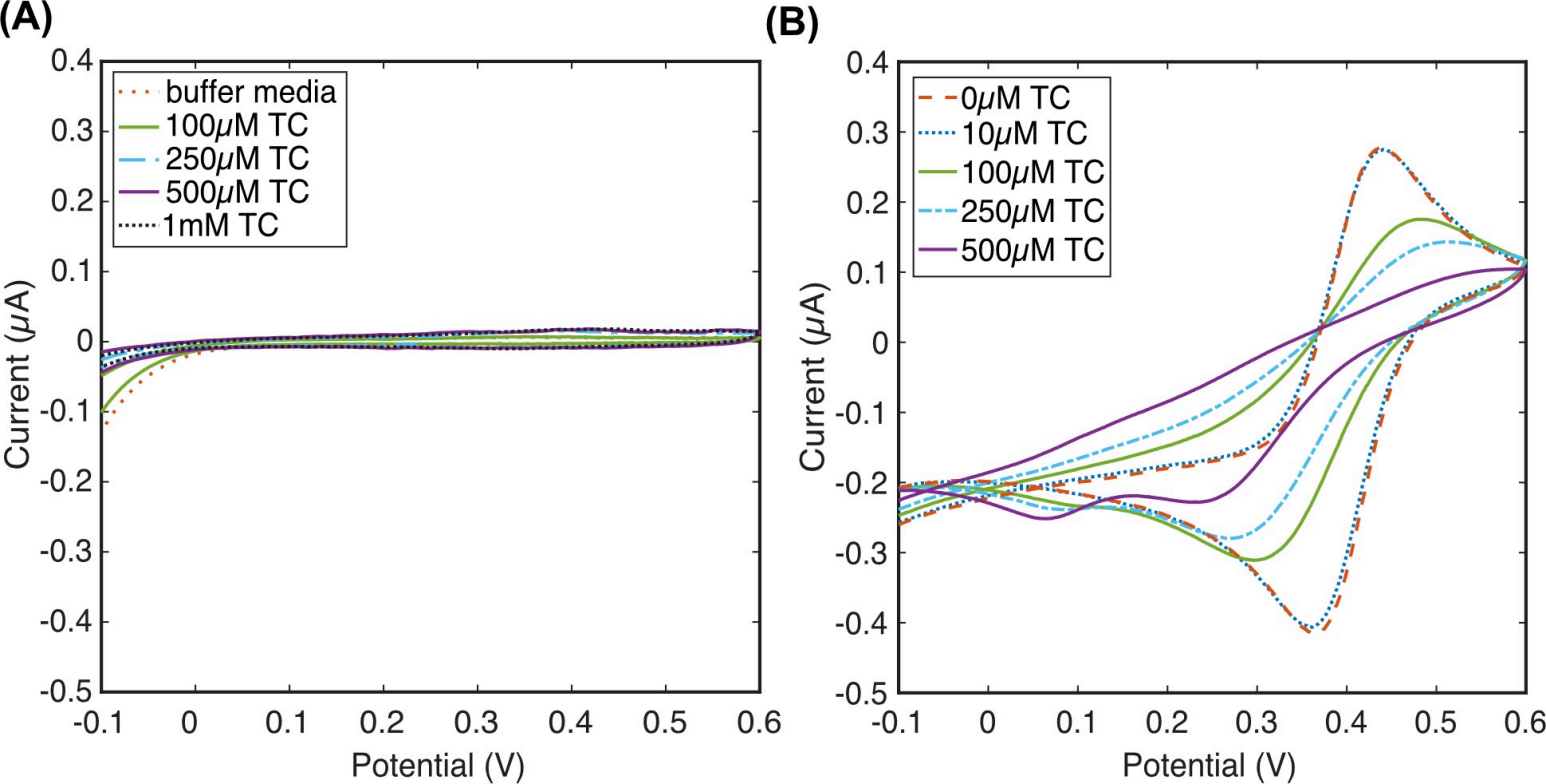
Electrochemical characterisation of the interplay between Fe(III) and TC on gold electrodes in dH_2_O containing 10mM KCl adjusted to pH 2.0. (A) CV of buffer media only and TC in buffer without Fe(III) showing no redox activity; (B) CV of 1 mM Fe(III) upon addition of TC at a range of concentrations.

TC is highly soluble at low pH because it is fully protonated and adopts a twisted conformation that allows metal binding [51–54]. Under these conditions, we explored the redox characteristics of TC, in the absence of Fe(III) to establish electrochemical activity using CV (Fig 4A). This showed that TC is not redox active across a wide range of concentrations from 100 μM to 1 mM between a potential range of -0.4 and +0.6 V vs. Ag/AgCl QRE. TC is reported to be redox active in the presence of anionic surfactant, such as sodium dodecylsulfate [52, 55]. A notable exception to this is Cánovas et al., who found that TC is redox active at +0.8 V on a carbon screen printed electrode in a solution with pH 2.0. This activity is believed to be due to differences in the oxidation and reduction potentials at carbon, vs AuNS electrode surfaces [53]. In this work, to express this, Au/AuNS electrodes offer analysis on gold material. A contradiction to that was found in a report by Casella et el., who scanned TC on a crystalline gold surface [56]. However, the media used was perchloric acid (HClO_4_) which has a cleaning effect on the gold electrode and the CV completely overlaps with the characteristic gold hydroxide species oxidation peak during gold electrode electrochemical cleaning that increases with each consecutive cycle, hence, it cannot be concluded that TC is actually detectable in this configuration [57].

Once it was established that TC was not redox activity on AuNS electrode system, we then explored the redox behaviour of free Fe(III) in isolation. A reversible redox couple was observed with a redox potential of 422 mV vs. Ag/AgCl QRE (Fig 4B). This aligns with the standard potential for the *Fe*(*III*)+*e*⇌*Fe*(*II*) reaction of 770 mV vs. NHE (See S2 Fig for derivation) [58]. The peak-to-peak separation (Δ*E*_p_) of the anodic and cathodic peak potentials of Fe(III) was 70 mV, which is larger than the theoretical value of 59 mV, probably due to a slower electron transfer between the solution and the electrode [59]. In addition, in majority electrochemical studies, the electrode-solution behaviour is not controlled by the kinetics of the electron transfer. Factors such as keeping the working electrode surface absolutely clean and well-defined or solution impurities adsorbing onto it can also contribute to reactions not being perfectly reversible [58].

Increasing concentrations of TC were added to electrolyte solutions containing 1 mM of Fe(III) and 10 mM KCl as the supporting electrolyte (Fig 3B) in order to explore the electrochemical impact of iron chelating TC on the *Fe*(*III*)+*e*⇌*Fe*(*II*) redox reaction. Initially, three scans were performed on each electrode to test the consistency of the measurements, where the repeat scans indicated that the electrode properties did not change while measuring the TC-Fe(III) complex solution at 1:2 ratio (S3 Fig). Under the pH 2.0 conditions applied here, the colour of the solutions immediately changed (S4B Fig). From TC concentrations of 10μM, a drop in the magnitude of the *Fe*(*III*)+*e*⇌*Fe*(*II*) oxidation and reduction peaks was observed, with complete loss of redox peaks at an TC:Fe(III) 2 mM to 1 mM, respectively, or ratio of 2:1 (Fig 4B). Instead, two new reduction peaks appeared at 0.06 V and 0.24 V (vs. Ag/AgCl QRE). The new peaks are believed to be caused by Fe(III) ions chelated by TC molecules resulting in a soluble complex with a much smaller current compared to Fe(III) alone. Furthermore, the reduction peaks are not matched by a corresponding oxidation peak, indicating these reactions are irreversible. When Fe(III) is complexed with TC immediately upon addition, the electron transfer is slower than the mass transfer and Fe(III) is reduced at a more negative potential. This was confirmed through UV-Vis spectroscopy [43], where a new peak emerges at 444 nm upon combination of TC and Fe(III) and the measured absorbance increases linearly when more TC was added to constant ferric ions content (S5 Fig).

**Fig 5:**
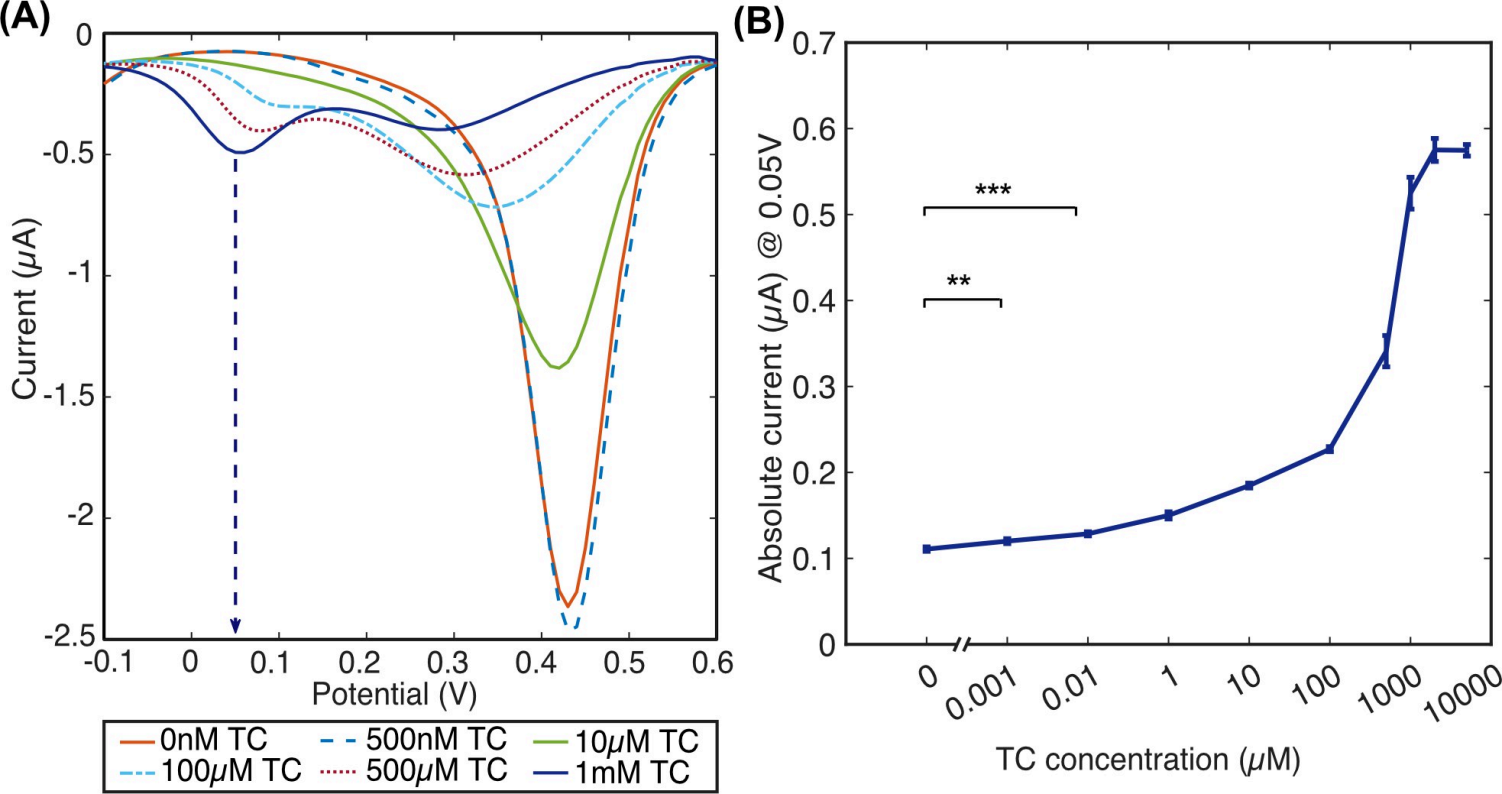
Electrode response to increasing TC concentrations in the presence of 1 mM Fe(III). (A) is DPV example of 1 mM Fe(III) when TC is added at an increasing range of concentrations (indicated in the plot legend) scanned on plasma-treated Au/AuNS electrode, where an arrow points the potential of + 50 mV that belongs to the newly formed peak; (B) relationship between the measured absolute current (at + 50 mV) vs. TC concentrations added to 1 mM Fe(III); *n =* 8, error bars +/- 1 SD, *** P < 0.001, ** P < 0.01 Student’s paired t-test.

CV measurement is valuable for qualitative analysis of the processes underway at an electrode surface. However, the non-faradaic charging of the electrode during a CV sweep can make it difficult to measure faradaic redox reactions. Alternatively, DPV can provide a better basis for analysis of redox reactions. DPV measurements show the peaks seen in the CV more clearly, measurable peak from a TC-Fe(III) ratio of 1:1 (Fig 5A) slightly sooner in the voltammogram at + 50 mV (vs. Ag/AgCl QRE). The peak potential was seen to change as a response to the addition of TC at 100 μM to 1 mM and may have been caused by a small variation in the pH of the solution due to TC addition or due to instability of the Ag/AgCl QRE system. Even though in pH < 3.5 Fe(III) is soluble and stable and is not expected to form complexes with Cl ions, a reaction might have occurred that influenced that potential shift [60]. In further development, this could be circumvented through the use of an alternative reference system, modification of the electrode system with a doped membrane, or a different electrochemical detection method that is less sensitive to small changes in redox potential.

Interestingly, the sensitivity of the system increases when measurements are performed with a lower concentration of Fe(III). Using a lower concentration of Fe(III), we tested the sensitivity of the electrochemical system, under Fe(III) concentration of 500 μM. This shows a similar response of the TC-Fe(III) complex, the newly emerged peak as a result of titrations of TC into solution containing Fe(III) reaches a maximum value at an TC:Fe(III) 1 mM to 500 μM concentrations, respectively or ratio 2:1 (Fig 5B).

In these conditions the related peak at + 50 mV does not increase further even at higher TC concentrations added to 500 μM Fe(III). This suggests a saturation of the complex formation and is consistent with two separate reports by Zhou et al. and Bagheri et al. that suggest the stoichiometry of TC-Fe(III) complex is stable at 2:1 ratio at acidic pH [40, 43]. This further confirms the proposed complex structure in Fig 1 of an Fe(III) locked at the two 1,3-diketone groups of two TC molecules. The previously suggested dimethylamine group site for metal binding is supposedly not used in the complexation with Fe(III) at these conditions and remains open for complexation with other metal ions such as Mg^2+^, Cu^2+^, Co^2+^, Al^3+^, Pt^2+^ as proposed by Pulicharla et al. [35].

All tetracycline antibiotics share the same naphthalene core but have different outer groups. Thus, the metal chelation property using the same procedure was also investigated using OTC where similar performance was observed with the exception that the potential of the complex peak was observed at 0.03 V (vs. Ag/AgCl QRE) (S6 Fig). The form of the OTC used in this work was a hydrochloride salt. The addition of even a trace concentration of hydrochloride in our system could have caused that slight shift in the potential of the complex peak. Nonetheless, this experiment has further confirmed the utilisation of this unique metal chelation property of TCs for their detection.

EDTA is a well characterised metal chelator with higher affinity towards Fe(III) compared to TC. The complex was tested with the addition of EDTA to further explore the interplay between TC and Fe(III). The EDTA-Fe(III) complex on its own has been reported to be reversible and also rapidly forms in solutions due to the high affinity of EDTA to metal ions [61, 62], with reversible redox activity, at -0.05 V vs. Ag/AgCl [63]. In this analysis, upon addition of EDTA to TC-Fe(III) complex, a redox peak was observed at -0.09 V (vs. Ag/AgCl QRE) in DPV measurements. The measured current of the peak consequently increases with further addition of EDTA which causes the TC-Fe(III) peak to decrease until it fully disappears. Based on these titration measurements, the amount of EDTA required to break the TC-Fe(III) complex and fully pull Fe(III) away into its own complex was found to be 2.5 mM. CV and DPV voltammograms can be seen in the S7 Fig.

### Selectivity testing against potential interferents in buffer

**Fig 6:**
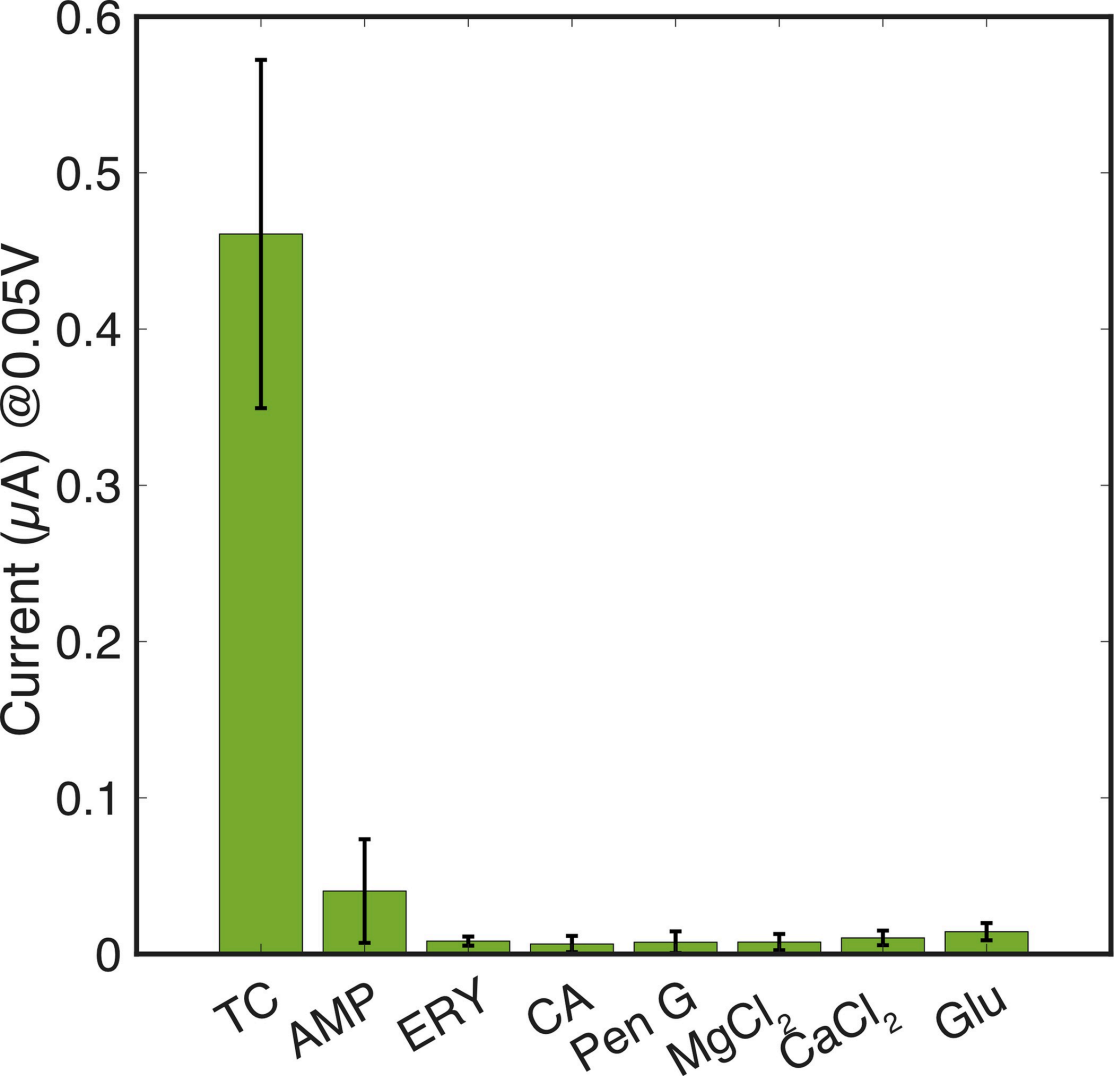
Selectivity test performed on modified electrodes showing the current response measured at the TC-Fe(III) complex peak potential (+ 50 mV) in the presence of 250 μM tetracycline first compared to when TC is not present along with other potential interferents (*n =* 8, error bars +/- 1 SD). TC = tetracycline; AMP = ampicillin; CA = chloramphenicol; Pen G = penicillin G; ERY = erythromycin; MgCl_*2*_ = magnesium chloride; CaCl_*2*_ = calcium chloride; Glu = glucose.

The specificity of this TC direct detection method was assessed in more representative samples. Calcium and magnesium concentration in milk, are report to be 2.5 mM and 250 μM, respectively [64–66]. Glucose concentration in cow’s milk is between the range of 190 and 260 μM, depending on breed [67]. Beta-lactam, chloramphenicol and erythromycin antibiotics are also used to treat infection in cattle. Therefore, ampicillin and penicillin G, chloramphenicol, erythromycin, glucose, MgCl_2_ and CaCl_2_ were explored as potential interferents. Each compound was added separately at 250 μM concentration to 1 mM Fe(III) in measurement buffer (Fig 6). Using the peak detection function available in the PS Trace software, the current magnitude at + 50 mV of each measurement was compared to the current magnitude of TC-Fe(III), when TC is at the same concentration. Of the tested interferents, no significant difference to the electrochemical response of the TC-Fe(III) complex was seen (*P < 0*.*001 Student’s paired t-test*, *n* = 8, Fig 6). In a separate test, TC-Fe(III) was complexed at a 1:2 ratio, respectively, and all interferences were added to the same solution at 250 μM concentration. The measured current at + 50 mV did not change when in the presence of the other potential interfering molecules as it can be seen in S8 Fig.

### Direct detection following milk preparation

In addition to the interferents described above, proteins and lipids within milk have the potential to foul the electrode surface inhibiting the reaction, and to compete with TC for Fe(III), thus reducing assay sensitivity [68]. For example, untreated cow’s milk contains lactoferrin, an iron-binding protein [69]. Initial testing of untreated milk spiked with TC and Fe(III) was performed, however, TC could not be detected due to matrix effect being induced that resulted in a complete loss of signal. Subsequently, removal of these interferent molecules from the milk matrix was carried before introducing Fe(III) ions to increase signal to noise. Sample preparation was carried out consisting of acidic protein precipitation and removal of the protein pellet via filtration. Further dilution of the samples was completed prior to adding TC and Fe(III) and finally the solutions were measured on the AuNS electrodes. As mentioned, for TC drug residue quantification in milk using HPLC, EDTA is typically added first to break TC-Mg and TC-Ca complexes. In this work, samples were processed first prior to spiking with TC and Fe(III) to avoid addition of EDTA and possible competition with TC for added Fe(III) ions.

**Fig 7:**
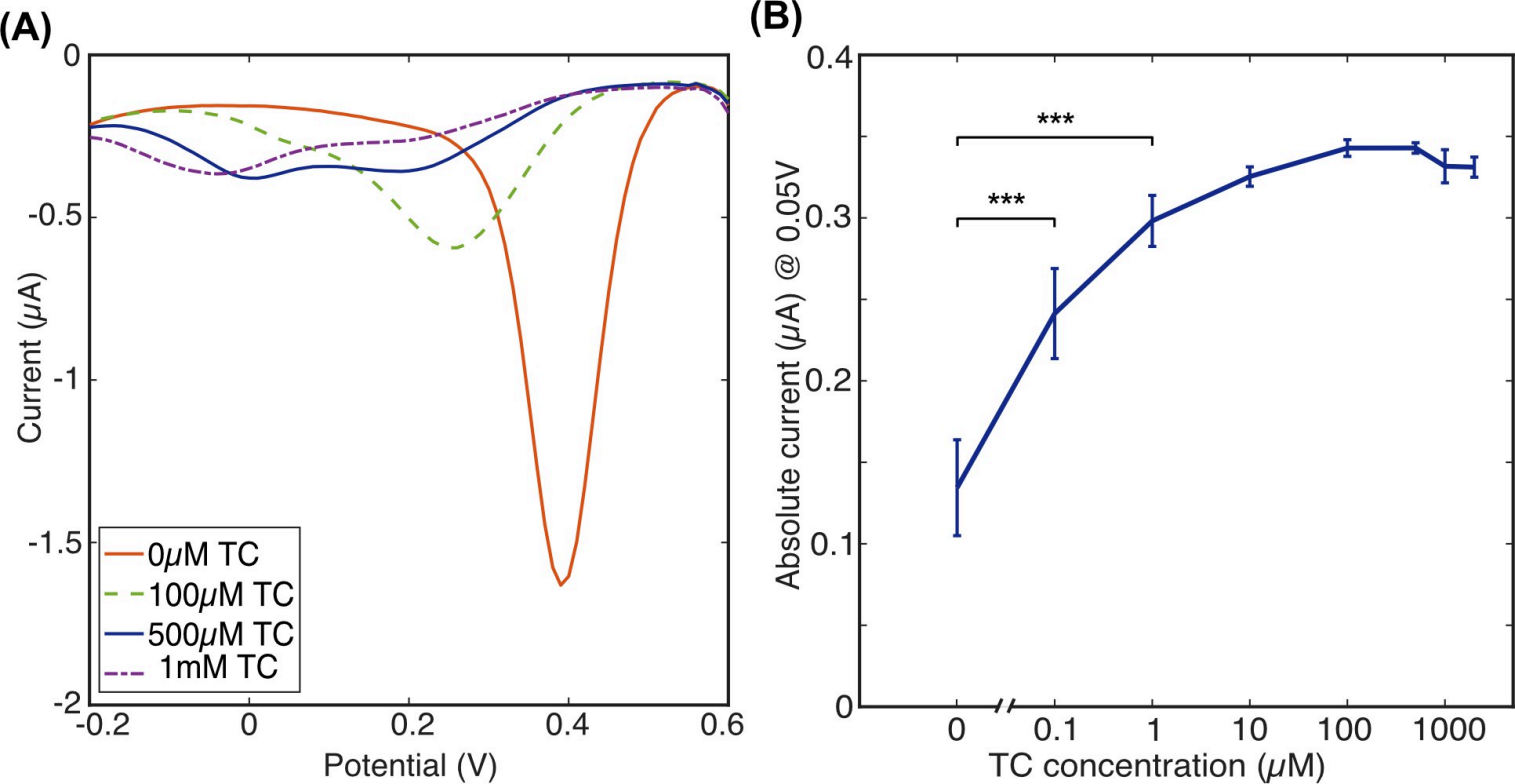
Detection of TC-Fe(III) complex in 1:100 whole milk samples; (A) DPV plot of 1 mM Fe(III) in milk and upon addition of TC at a range of concentrations; (B) the absolute current measured at + 50 mV (vs. Ag/AgCl QRE) when TC was added at a range of concentrations in addition to 1 mM Fe(III); *n =* 8 error bars +/- 1 SD; t-test was performed, where obtained p-values were found to equate ***P < 0.001.

As Fig 7 shows, the TC-Fe(III) current peak was still observed in these samples, but with a drop in peak current magnitude (absolute current 0.34 μA compared to maximum current measured in buffer being 0.57 μA). Also, there is an apparent saturation when TC:Fe(III) are at 500 μM to 1 mM, respectively, (or ratio 1:2), which was not seen in the measurements in buffer conditions, where saturation occurred at 1 mM TC added to 500 μM Fe(III), or ratio 2:1, respectively. This could be explained by residual proteins or lipids in the milk matrix that could have fouled the electrode surface during analysis. Hence, a drop in the current is also observed after 500 μM TC in the milk samples, which was not seen in the measurements in buffer conditions. In addition, with Ca^2+^ ion present in much higher concentration than 250 μM, it is likely that it had complexed with TC prior to the addition of Fe(III). Therefore, a multi-stage sample preparation is required to remove proteins and ions from milk prior to addition of Fe(III) for the detection of TC which would form the subject for a future study.

### Analytical capabilities of sensor system

The limits of detection (LOD) and quantification (LOQ) were calculated for both the electrochemical and UV-Visible spectroscopy methods using the current at complex peak potential (+ 50 mV) and absorbance (444 nm), using eqt (1) and (2) [70]. Where *σ*is the standard deviation of the measurement with no TC in the sample and *S* is the slope of the calibration curve with increasing concentrations of TC up to 100 μM.,

$$\mathrm{LOD}=3.3(\frac{\sigma }{S})$$ (Eq 1)

$$\mathrm{LOQ}=10(\frac{\sigma }{S})$$ (Eq 2)

Obtained values for measurements in buffer at pH 2.0 and processed milk at pH 2.0 are shown in Table 1. The linearity of TC concentration added to 1 mM Fe(III), correlation coefficient (R^2^) as well as precision, accuracy and reproducibility (*n* = 3, at 500 μM TC added to 1 mM Fe(III)) expressed as R.S.D. (%) of both methods were also included in Table 1.

**Table 1:** Limits of quantification and detection, linearity, accuracy and precision for TC-Fe(III) complex using electrochemical and UV-Vis measurements in measurement buffer at pH 2.0 and in deproteinised, filtered milk diluted 1:100 with measurement buffer pH 2.0.

Method/Parameter	Electrochemical method		UV-Vis spectroscopy	
	buffer	milk	buffer	milk
**LOD (μM)**	0.345	0.931	5.89	7.67
**LOQ (μM)**	1.05	2.82	17.65	23.26
**Linearity (μM)**	0.1–100	1–100	100–1000	100–1000
**R** ^**2**^	0.965	0.715	0.952	0.926
**R.S.D. (%)**	2.60	4.49	0.13	0.27

Limits for TC residues in milk are 225 nM, and TC has been detected in environmental samples between 58 nM and 630 nM. The LOD reported here for electrochemical detection in buffer of 345 nM is of relevance within environmentally detected concentrations. In milk, the direct detection approach was less sensitive and approximately four times above the MRL of 225 nM. Future work will therefore focus upon more robust sample preparation techniques that will increase the removal of residues from milk with the aim of bringing the LOD down. Milk is a notoriously difficult matrix to analyse, sample preparation strategies are required to make the approach described here effective in milk.

It was noted that the linear range of the sensor developed here was less than 100 uM for all sample types. To the best of our knowledge, the highest reported concentration of TC residue has been found in a farm manure sample at 20 mg/L [22], equating to 45 μM. This is within the 100 μM linear range of the sensor developed here, suggesting that beyond milk and food residue testing, there are several useful surveillance applications for this approach.

Interestingly, the results reported here show 17 times greater sensitivity than UV-visible spectroscopy for both buffer and extracted milk samples. This suggests that electrochemical detection of TC residues for in-field applications might be appropriate than optical measurement techniques based upon UV-visible spectroscopy. Furthermore, electrochemical instrumentation can be packaged into small devices at low cost.

In contrast to other reported studies (Table 2), the direct detection electrode and approach describe here has comparable performance to direct detection techniques. The most sensitive methods report use aptameric based biosensors for the affinity based capture of TC at the electrode surface [71, 72]. Whilst these represent outstanding sensitivities, they have the drawback of electrode preparation, storage and aptamer folding prior to use. In a commercial context, these would add cost and time to the measurement assay making their application more challenging.

**Table 2:** Comparison of this work’s LOD in buffer to LODs of other electrochemical methods for detection of tetracyclines. WE = working electrode; RE = reference electrode; TC = tetracycline; DMC = demeclocycline; SPGE-AuNP-Cys = screen-printed gold electrode covered in gold nanoparticles and cysteine SAM; BDD = boron-doped diamond electrode; SPCE = screen-printed carbon electrode; QRE = quasi-reference electrode; MIOPPy = molecularly imprinted polypyrrole; GCE-MWCNTs = glassy carbon electrode modified with multi-walled carbon nanotubes; SWV = square wave voltammetry; SW-AdSV = square wave adsorptive stripping voltammetry; DPV = differential pulse voltammetry; MIP = molecularly imprinted polymer.

Analyte	WE and RE materials	Electrochemical technique	Transduction principle	LOD	Matrix	Ref
TC	Au/AuNS (QRE: Ag/AgCl)	DPV	Direct detection	0.306 μM	buffer (pH 2.0)	This work
				0.931 μM	milk spiked post sample prep	
TC	SPGE-AuNP-Cys (RE: Ag)	SWV	Direct detection	0.52 μM	milk	[51]
DMC	BDD (RE: Ag/AgCl)	SW-AdSV	Direct detection in the presence of surfactants	2.30 μM	buffer (pH 2.0)	[52]
				0.48 μM	buffer (pH 9.0)	
TC	SPCE (QRE: Ag)	SWV	Direct detection	4.15 μM	buffer (pH 9.0)	[53]
TC	MIOPPy-AuNP/SPCE (RE: Ag/AgCl)	DPV	MIP in the presence of surfactant	0.65 μM	buffer (pH 1.5)	[55]
TC	SPGE (RE: Ag)	SWV	aptamer	0.010 μM	buffer (pH 7.6)	[71]
TC	GCE-MWCNTs (RE: Ag/AgCl)	DPV	aptamer	0.005 μM	buffer (pH 7.6)	[72]

## Conclusion

This study demonstrates the electrochemical characterisation and detection of TC in simple buffer and milk samples by exploiting the redox properties of the TC-Fe(III) complex. A TC-Fe(III) complex was formed in acidic aqueous conditions and was directly detected electrochemically on a plasma-treated Au/AuNS platform. The selectivity of this electrochemical method was also examined and the TC-Fe(III) complex was not interfered by other potentially present in the complex matrices containing antibiotics, glucose and ions. In summary, this study describes a reliable alternative analytical technique for TC detection. Future work will focus upon sample preparation and increasing sensor performance within milk samples.

## Supporting information

S1 Fig SEM images at magnification 1000x, scale indicated on image, where (A) edge of bare gold electrode; (B) edge of electrode after electrodeposition of AuNS in 5mM HAuCl_4_ solution prepared in 0.5M H_2_SO_4_ using chronoamperometry.
(TIFF)

S2 Fig Ag/AgCl pseudo reference electrode (QRE) vs Ag/AgCl external reference electrode. The FlexMedical electrode chips we have employed in this reported analysis consist of eight gold working (1 mm in diameter), a gold common counter electrode and a common Ag/AgCl pseudo reference electrode (QRE). To test the potential difference of our QRE to Ag/AgCl RE, we isolated the common QRE from the chip and connected an external Ag/AgCl (1M KCl) reference. (A) CV measurements in 1 mM FeCl_3_ in pH 2.0 media adjusted with 1 M HCl containing 20 mM KCl were taken with both connections separately and the difference in the oxidation peak potential was calculated as 170 mV; (B) further confirmation of the result by running an open circuit potentiometric measurement for 300 seconds at a time interval of 1 second by connecting the common QRE outlet as the working electrode and the external Ag/AgCl RE to the chip. This resulted in a steady potential of 182 mV over the duration of the analysis.
(TIFF)

S3 Fig CV of repeated measurements (*n* = 3) on Au/AuNS electrode of TC-Fe(III), where TC at 500 μM was added to 1 mM Fe(III) to form a complex. The consistency of the three measurements indicated that the electrode properties did not change.
(TIFF)

S4 Fig Pictures of (A) Au working electrodes after electrodeposition of AuNS, where newly formed Au layer can be observed physically; (B) A picture of solutions already containing 1mM FeCl_*3*_ upon addition of TC at a range of concentrations indicated on image, where colour changed with increasing TC concentration.
(TIFF)

S5 Fig UV-Vis analysis of TC-Fe(III) solutions in pH 2.0; (A) UV-Vis spectrum of TC (100 μM) and FeCl_*3*_ (1 mM) separately and in a combination scanned between 380 and 500 nm, where a newly emerged peak appears at 444 nm only upon complex formation; (B) Proportional relationship between TC concentration range added to 1 mM Fe(III) vs. measured UV absorbance at 444 nm, resulting in a sigmoidal curve with saturation of the TC-Fe(III) complex at 2:1 ratio.
(TIFF)

S6 Fig Electrode response to increasing OTC concentrations in the presence of 1 mM Fe(III), where (A) relationship between the measured absolute current (at 0.03 V) vs. OTC concentrations added to 1 mM Fe(III) resulting in a sigmoidal curve with error bars added that stand for the standard deviation between 8 replicate electrodes; t-test was performed, where obtained p-values were found as ***p < 0.001; (B) is DPV example of 1 mM Fe(III) when OTC is added at an increasing range of concentrations (indicated in the plot legend) scanned on plasma-treated Au/AuNS electrode.
(TIFF)

S7 Fig Stability of the TC-Fe(III) complex: EDTA complexes Fe(III) from TC and results in a fingerprint (scanned on Au/AuNS electrodes, *n* = 8) of the three compounds present simultaneously in a solution; (A) Upon addition of EDTA to TC-Fe(III) complex, a reversible redox couple was observed as oxidation peak at -0.07 V and reduction peak at -0.15 V; (B) The redox peak observed in DPV measurements was at -0.09 V (vs. Ag/AgCl QRE). This is consistent with the reported potential of the EDTA-Fe(III) redox couple by Allcorn et al. when considered in the context of the Ag/AgCl QRE used here and the scan rate of 50 mV/s [61]. The measured current of the peak consequently increases with further addition of EDTA which causes the TC-Fe(III) peak to decrease until it fully disappears from the DPV spectrum. This indicates that EDTA has pulled the TC-Fe(III) complexed iron apart, in addition to chelating all remaining free iron in the solution. Since TC is not electrochemically active on its own, only the peak that belongs to EDTA-Fe(III) remained in the scanned DPV spectrum. Based on these titration measurements, the amount of EDTA required to break the TC-Fe(III) and fully pull Fe(III) away into its own complex was found to be 2.5 mM or 1:2.5 ratio of TC:EDTA co-existing in a solution, respectively.
(TIFF)

S8 Fig Interference test performed on modified electrodes showing the current response measured at the TC-Fe(III) complex peak potential (+ 50 mV) when 500 μM TC were complexed with 1 mM Fe(III) in the presence of respective potential interferents at 250 μM concentration (n = 8, error bars +/- 1 SD). TC = tetracycline; AMP = ampicillin; CA = chloramphenicol; Pen G = penicillin G; ERY = erythromycin; MgCl_2_ = magnesium chloride; CaCl_2_ = calcium chloride.
(TIFF)

S1 Dataset (XLSX)

## References

[1] Sachi S, Ferdous J, Sikder H, Azizul SM, Hussani K. Antibiotic residues in milk: Past, present, and future. J Adv Vet Anim Res 2019;6:315–32. doi: 10.5455/javar.2019.f350 31583228 PMC6760505

[2] Baghani A, Mesdaghinia A, Rafieiyan M, Dallal MMS, Douraghi M. Tetracycline and ciprofloxacin multiresidues in beef and chicken meat samples using indirect competitive ELISA. J Immunoassay Immunochem 2019;40:328–42. doi: 10.1080/15321819.2019.1597735 30945975

[3] 14:00–17:00. ISO/TS 26844:2006. ISO n.d. https://www.iso.org/cms/render/live/en/sites/isoorg/contents/data/standard/04/38/43819.html (accessed November 5, 2021).

[4] 14:00–17:00. ISO 18330:2003. ISO n.d. https://www.iso.org/cms/render/live/en/sites/isoorg/contents/data/standard/03/34/33421.html (accessed November 5, 2021).

[5] Kurjogi M, Mohammad YHI, Alghamdi S, Abdelrahman M, Satapute P, Jogaiah S. Detection and determination of stability of the antibiotic residues in cow’s milk. PLoS ONE 2019;14. doi: 10.1371/journal.pone.0223475 31600289 PMC6786530

[6] Hennart SLA, Faragher J, Boison J, Agin J, Mitchell M. Validation of the delvotest SP NT DA. J AOAC Int 2012;95:252–60. 10.5740/jaoacint.11-138. 22468368

[7] Raykova MR, Corrigan DK, Holdsworth M, Henriquez FL, Ward AC. Emerging Electrochemical Sensors for Real-Time Detection of Tetracyclines in Milk. Biosensors n.d.;11:232. doi: 10.3390/bios11070232 34356702 PMC8301834

[8] Chopra I, Roberts M. Tetracycline Antibiotics: Mode of Action, Applications, Molecular Biology, and Epidemiology of Bacterial Resistance. Microbiol Mol Biol Rev 2001;65:232–60. doi: 10.1128/MMBR.65.2.232-260.2001 11381101 PMC99026

[9] Johnson R, Adams J. The ecology and evolution of tetracycline resistance. Trends Ecol Evol 1992;7:295–9. doi: 10.1016/0169-5347(92)90226-2 21236038

[10] Warner AJ, Hathaway-Schrader JD, Lubker R, Davies C, Novince CM. Tetracyclines and bone: Unclear actions with potentially lasting effects. Bone 2022;159:116377. doi: 10.1016/j.bone.2022.116377 35248788 PMC9035080

[11] Boisseau J, Moretain JP. Drug excretion by the mammary gland. In: Ruckebusch Y, Toutain P-L, Koritz GD, editors. Vet. Pharmacol. Toxicol., Dordrecht: Springer Netherlands; 1983, p. 193–202. 10.1007/978-94-009-6604-8_18.

[12] Ziv G, Sulman FG. Absorption of Antibiotics by the Bovine Udder. J Dairy Sci 1975;58:1637–44. doi: 10.3168/jds.S0022-0302(75)84762-X 1194466

[13] Sarmah AK, Meyer MT, Boxall ABA. A global perspective on the use, sales, exposure pathways, occurrence, fate and effects of veterinary antibiotics (VAs) in the environment. Chemosphere 2006;65:725–59. doi: 10.1016/j.chemosphere.2006.03.026 16677683

[14] EMA. Determination of withdrawal periods for milk—Scientific guideline. Eur Med Agency 2018. https://www.ema.europa.eu/en/determination-withdrawal-periods-milk-scientific-guideline (accessed December 19, 2022).

[15] Siwicka C. Maximum Residue Limits in Great Britain 2021.

[16] Khurana P, Pulicharla R, Kaur Brar S. Antibiotic-metal complexes in wastewaters: fate and treatment trajectory. Environ Int 2021;157:106863. doi: 10.1016/j.envint.2021.106863 34534786

[17] Batt AL, Aga DS. Simultaneous analysis of multiple classes of antibiotics by ion trap LC/MS/MS for assessing surface water and groundwater contamination. Anal Chem 2005;77:2940–7. doi: 10.1021/ac048512+ 15859614

[18] Jacobsen AM, Halling-Sørensen B, Ingerslev F, Hansen SH. Simultaneous extraction of tetracycline, macrolide and sulfonamide antibiotics from agricultural soils using pressurised liquid extraction, followed by solid-phase extraction and liquid chromatography-tandem mass spectrometry. J Chromatogr A 2004;1038:157–70. doi: 10.1016/j.chroma.2004.03.034 15233531

[19] Hamscher G, Sczesny S, Höper H, Nau H. Determination of persistent tetracycline residues in soil fertilized with liquid manure by high-performance liquid chromatography with electrospray ionization tandem mass spectrometry. Anal Chem 2002;74:1509–18. doi: 10.1021/ac015588m 12033238

[20] Ben W, Zhu B, Yuan X, Zhang Y, Yang M, Qiang Z. Occurrence, removal and risk of organic micropollutants in wastewater treatment plants across China: Comparison of wastewater treatment processes. Water Res 2018;130:38–46. doi: 10.1016/j.watres.2017.11.057 29197755

[21] Yang S, Cha J, Carlson K. Simultaneous extraction and analysis of 11 tetracycline and sulfonamide antibiotics in influent and effluent domestic wastewater by solid-phase extraction and liquid chromatography-electrospray ionization tandem mass spectrometry. J Chromatogr A 2005;1097:40–53. doi: 10.1016/j.chroma.2005.08.027 16298184

[22] Winckler C, Grafe A. Transfer of veterinary drugs and pharmacologically-active feed additives into soil with special consideration of tetracyclines–executive summary. J Soils Sediments 2001;1:58–62. 10.1007/BF02986471.

[23] Scaria J, Anupama KV, Nidheesh PV. Tetracyclines in the environment: An overview on the occurrence, fate, toxicity, detection, removal methods, and sludge management. Sci Total Environ 2021;771:145291. doi: 10.1016/j.scitotenv.2021.145291 33545482

[24] Wang L, Zhang C, Wu F, Deng N, Glebov EM, Bazhin NM. Determination of hydroxyl radicals from photolysis of Fe(III)-pyruvate complexes in homogeneous aqueous solution. React Kinet Catal Lett 2006;89:183.

[25] Kuma K, Nishioka J, Matsunaga K. Controls on iron(III) hydroxide solubility in seawater: The influence of pH and natural organic chelators. Limnol Oceanogr 1996;41:396–407. 10.4319/lo.1996.41.3.0396.

[26] Millero FJ, Yao W, Aicher J. The speciation of Fe(II) and Fe(III) in natural waters. Mar Chem 1995;50:21–39. 10.1016/0304-4203(95)00024-L.

[27] Huang F, Xin S, Mi T, Zhang L. Investigation on the transformation behaviours of Fe-bearing minerals of coal in O 2 /CO 2 combustion atmosphere containing H 2 O. RSC Adv 2021;11:10635–45. doi: 10.1039/d1ra00673h 35423589 PMC8695706

[28] Morgan JW, Anders E. Chemical composition of Earth, Venus, and Mercury. Proc Natl Acad Sci 1980;77:6973–7. doi: 10.1073/pnas.77.12.6973 16592930 PMC350422

[29] Stefánsson A. Iron(III) Hydrolysis and Solubility at 25°C. Environ Sci Technol 2007;41:6117–23. 10.1021/es070174h. 17937290

[30] Lee S, Kim WJ, Chung M. Enhanced electrochemical biosensing on gold electrodes with a ferri/ferrocyanide redox couple. Analyst 2021;146:5236–44. doi: 10.1039/d1an00952d 34378551

[31] Daum PH, Enke CG. Electrochemical kinetics of the ferri-ferrocyanide couple on platinum. Anal Chem 1969;41:653–6. 10.1021/ac60273a007.

[32] Alberti G, Zanoni C, Rovertoni S, Magnaghi LR, Biesuz R. Screen-Printed Gold Electrode Functionalized with Deferoxamine for Iron(III) Detection. Chemosensors 2022;10:214. 10.3390/chemosensors10060214.

[33] Zhu Y, Hu X, Pan D, Han H, Lin M, Lu Y, et al. Speciation determination of iron and its spatial and seasonal distribution in coastal river. Sci Rep 2018;8:2576. doi: 10.1038/s41598-018-20991-0 29416105 PMC5803190

[34] Carlotti B, Cesaretti A, Elisei F. Complexes of tetracyclines with divalent metal cations investigated by stationary and femtosecond-pulsed techniques. Phys Chem Chem Phys 2012;14:823–34. doi: 10.1039/c1cp22703c 22120200

[35] Pulicharla R, Das RK, Brar SK, Drogui P, Sarma SJ, Verma M, et al. Toxicity of chlortetracycline and its metal complexes to model microorganisms in wastewater sludge. Sci Total Environ 2015;532:669–75. doi: 10.1016/j.scitotenv.2015.05.140 26119381

[36] Grenier D, Huot M-P, Mayrand D. Iron-Chelating Activity of Tetracyclines and Its Impact on the Susceptibility of Actinobacillus actinomycetemcomitans to These Antibiotics. Antimicrob Agents Chemother 2000;44:763–6. doi: 10.1128/AAC.44.3.763-766.2000 10681353 PMC89761

[37] Wang H, Yao H, Sun P, Li D, Huang C-H. Transformation of Tetracycline Antibiotics and Fe(II) and Fe(III) Species Induced by Their Complexation. Environ Sci Technol 2016;50:145–53. doi: 10.1021/acs.est.5b03696 26618388

[38] Wang H, Yao H, Sun P, Pei J, Li D, Huang C-H. Oxidation of tetracycline antibiotics induced by Fe(III) ions without light irradiation. Chemosphere 2015;119:1255–61. doi: 10.1016/j.chemosphere.2014.09.098 25460769

[39] Korać Jačić J, Milenković MR, Bajuk-Bogdanović D, Stanković D, Dimitrijević M, Spasojević I. The impact of ferric iron and pH on photo-degradation of tetracycline in water. J Photochem Photobiol Chem 2022;433:114155. 10.1016/j.jphotochem.2022.114155.

[40] Bagheri A. Thermodynamic Studies of Metal Complexes of Tetracycline and its Application in Drug Analysis. Pharm Chem J 2015;48:765–9. 10.1007/s11094-015-1190-3.

[41] Zaitoun MA, Lin CT. Chelating Behavior between Metal Ions and EDTA in Sol−Gel Matrix. J Phys Chem B 1997;101:1857–60. 10.1021/jp963102d.

[42] Albert A, Rees CW. Avidity of the Tetracyclines for the Cations of Metals. Nature 1956;177:433–4. doi: 10.1038/177433a0 13309332

[43] Zhou D, Wang J, Hou L, Xu J, Zhao Y. Photochemistry of Fe(III)-Tetracycline Complexes in Aqueous Solution under UV Irradiation. 2012 Third Int. Conf. Digit. Manuf. Autom., 2012, p. 608–11. 10.1109/ICDMA.2012.144.

[44] Ghandour MA, Azab HA, Hassan A, Ali AM. Potentiometric studies on the complexes of tetracycline (TC) and oxytetracyclin (OTC) with some metal ions. Monatshefte Für Chem Chem Mon 1992;123:51–8. 10.1007/BF01045296.

[45] Ogino H, Shimura M. Advances in Inorganic and Bioinorganic Mechanism 1986.

[46] George T, Brady MF. Ethylenediaminetetraacetic Acid (EDTA). StatPearls, Treasure Island (FL): StatPearls Publishing; 2022. 33351441

[47] Gu C, Karthikeyan KG. Interaction of Tetracycline with Aluminum and Iron Hydrous Oxides. Environ Sci Technol 2005;39:2660–7. doi: 10.1021/es048603o 15884363

[48] Wiberg E, Wiberg N, Holleman AF. Inorganic chemistry. San Diego; Berlin; New York: Academic Press; De Gruyter; 2001.

[49] Wan H, Sun Q, Li H, Sun F, Hu N, Wang P. Screen-printed gold electrode with gold nanoparticles modification for simultaneous electrochemical determination of lead and copper. Sens Actuators B Chem 2015;209:336–42. 10.1016/j.snb.2014.11.127.

[50] Paradowska E, Arkusz K, Pijanowska DG. Comparison of Gold Nanoparticles Deposition Methods and Their Influence on Electrochemical and Adsorption Properties of Titanium Dioxide Nanotubes. Materials 2020;13:4269. doi: 10.3390/ma13194269 32992707 PMC7578957

[51] Asadollahi-Baboli M, Mani-Varnosfaderani A. Rapid and simultaneous determination of tetracycline and cefixime antibiotics by mean of gold nanoparticles-screen printed gold electrode and chemometrics tools. Meas J Int Meas Confed 2014;47:145–9. 10.1016/j.measurement.2013.08.029.

[52] Allahverdiyeva S, Yardım Y, Şentürk Z. Electrooxidation of tetracycline antibiotic demeclocycline at unmodified boron-doped diamond electrode and its enhancement determination in surfactant-containing media. Talanta 2021;223:121695. doi: 10.1016/j.talanta.2020.121695 33303147

[53] Cánovas R, Sleegers N, van Nuijs ALN, De Wael K. Tetracycline Antibiotics: Elucidating the Electrochemical Fingerprint and Oxidation Pathway. Chemosensors 2021;9:187. 10.3390/chemosensors9070187.

[54] Wessels JM, Ford WE, Szymczak W, Schneider S. The Complexation of Tetracycline and Anhydrotetracycline with Mg2+ and Ca2+: A Spectroscopic Study. J Phys Chem B 1998;102:9323–31. 10.1021/jp9824050.

[55] Devkota L, Nguyen LT, Vu TT, Piro B. Electrochemical determination of tetracycline using AuNP-coated molecularly imprinted overoxidized polypyrrole sensing interface. Electrochimica Acta 2018;270:535–42. 10.1016/j.electacta.2018.03.104.

[56] Casella IG, Picerno F. Determination of Tetracycline Residues by Liquid Chromatography Coupled with Electrochemical Detection and Solid Phase Extraction. J Agric Food Chem 2009;57:8735–41. doi: 10.1021/jf902086y 19754048

[57] Ovchinnikova SN, Medvedev AZh. Desorption of octanethiol from gold electrode surface during its electrochemical cleaning. Russ J Electrochem 2015;51:287–93. 10.1134/S1023193515040084.

[58] Bard AJ, Faulkner LR. Electrochemical Methods: Fundamentals and Applications, 2nd Edition | Wiley. 2nd ed. n.d.

[59] Elgrishi N, Rountree KJ, McCarthy BD, Rountree ES, Eisenhart TT, Dempsey JL. A Practical Beginner’s Guide to Cyclic Voltammetry. J Chem Educ 2018;95:197–206. 10.1021/acs.jchemed.7b00361.

[60] Kaplan J, Ward DM. The essential nature of iron usage and regulation. Curr Biol 2013;23:R642–6. doi: 10.1016/j.cub.2013.05.033 23928078 PMC3928970

[61] Allcorn E, Nagasubramanian G, Pratt HD, Spoerke E, Ingersoll D. Elimination of active species crossover in a room temperature, neutral pH, aqueous flow battery using a ceramic NaSICON membrane. J Power Sources 2018;378:353–61. 10.1016/j.jpowsour.2017.12.041.

[62] Verma PS, Saxena RC, Jayaraman A. Cyclic voltammetric studies of certain industrially potential iron chelate catalysts. Fresenius J Anal Chem 1997;357:56–60. 10.1007/s002160050110.

[63] Bai Z-P, Nakamura T, Izutsu K. Enhanced voltammetric waves of iron(III)—EDTA at a chitin-containing carbon paste electrode and its analytical application. Electroanalysis 1990;2:75–9. 10.1002/elan.1140020114.

[64] Tessier H, Rose D. Calcium Ion Concentration in Milk. J Dairy Sci 1958;41:351–9. 10.3168/jds.S0022-0302(58)90927-5.

[65] Li Y, Corredig M. Calcium release from milk concentrated by ultrafiltration and diafiltration. J Dairy Sci 2014;97:5294–302. doi: 10.3168/jds.2013-7567 25022683

[66] Oh HE, Deeth HC. Magnesium in milk. Int Dairy J 2017;71:89–97. 10.1016/j.idairyj.2017.03.009.

[67] Faulkner A, Chaiyabutr N, Peaker M, Carrick DT, Kuhn NJ. Metabolic significance of milk glucose. J Dairy Res 1981;48:51–6. doi: 10.1017/s0022029900021440 7264010

[68] Lin P-H, Li B-R. Antifouling strategies in advanced electrochemical sensors and biosensors. Analyst 2020;145:1110–20. doi: 10.1039/c9an02017a 31916551

[69] Superti F. Lactoferrin from Bovine Milk: A Protective Companion for Life. Nutrients 2020;12:2562. doi: 10.3390/nu12092562 32847014 PMC7551115

[70] Gumustas M, A. Ozkan S. The Role of and the Place of Method Validation in Drug Analysis Using Electroanalytical Techniques. Open Anal Chem J 2011;5.

[71] Kim YJ, Kim YS, Niazi JH, Gu MB. Electrochemical aptasensor for tetracycline detection. Bioprocess Biosyst Eng 2010;33:31–7. doi: 10.1007/s00449-009-0371-4 19701778

[72] Zhou L, Li DJ, Gai L, Wang JP, Li YB. Electrochemical aptasensor for the detection of tetracycline with multi-walled carbon nanotubes amplification. Sens Actuators B Chem 2012;162:201–8. 10.1016/j.snb.2011.12.067.

