# Supplementary figures and images for: Towards direct detection of tetracycline residues in milk with a gold nanostructured electrode
Source: PLoS One. 2023 Jun 27;18(6):e0287824. doi: 10.1371/journal.pone.0287824 (PMC10298779; doi:10.1371/journal.pone.0287824)

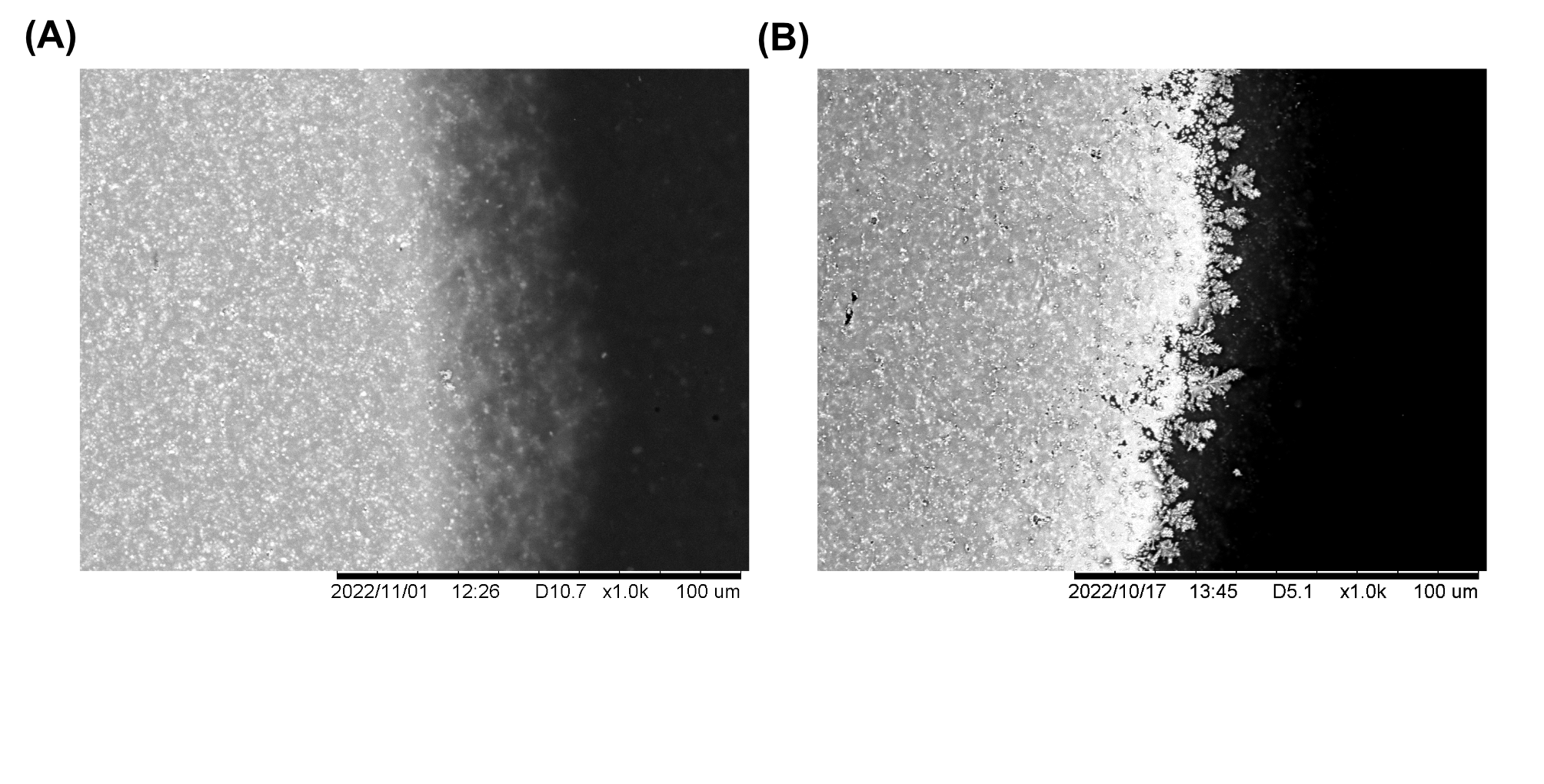

Supplement: S1 Fig — SEM images at magnification 1000x, scale indicated on image, where (A) edge of bare gold electrode; (B) edge of electrode after electrodeposition of AuNS in 5mM HAuCl4 solution prepared in 0.5M H2SO4 using chronoamperometry. (TIFF) [file pone.0287824.s001.tiff]

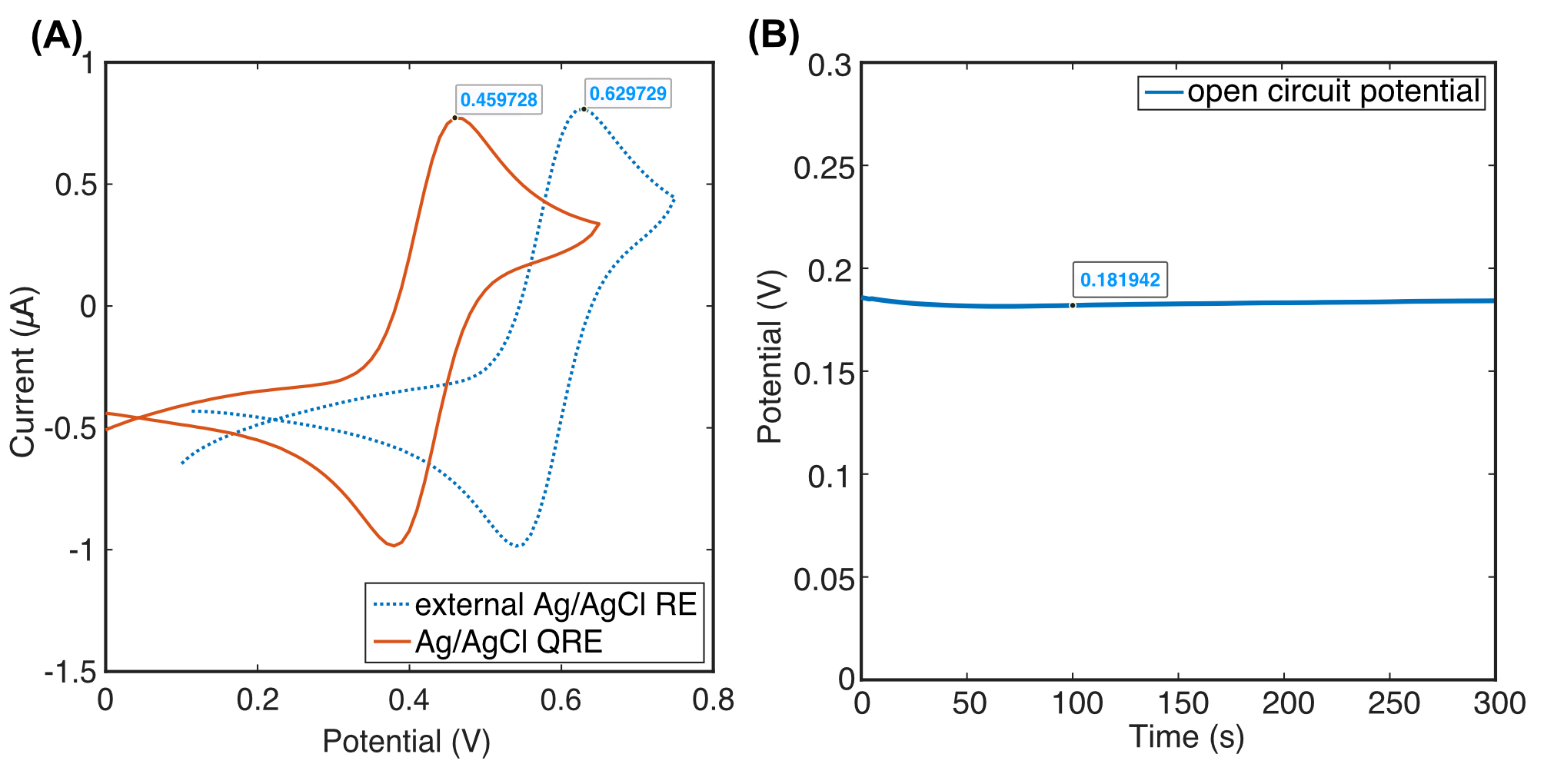

Supplement: S2 Fig — The FlexMedical electrode chips we have employed in this reported analysis consist of eight gold working (1 mm in diameter), a gold common counter electrode and a common Ag/AgCl pseudo reference electrode (QRE). To test the potential difference of our QRE to Ag/AgCl RE, we isolated the common QRE from the chip and connected an external Ag/AgCl (1M KCl) reference. (A) CV measurements in 1 mM FeCl3 in pH 2.0 media adjusted with 1 M HCl containing 20 mM KCl were taken with both connections separately and the difference in the oxidation peak potential was calculated as 170 mV; (B) further confirmation of the result by running an open circuit potentiometric measurement for 300 seconds at a time interval of 1 second by connecting the common QRE outlet as the working electrode and the external Ag/AgCl RE to the chip. This resulted in a steady potential of 182 mV over the duration of the analysis. (TIFF) [file pone.0287824.s002.tiff]

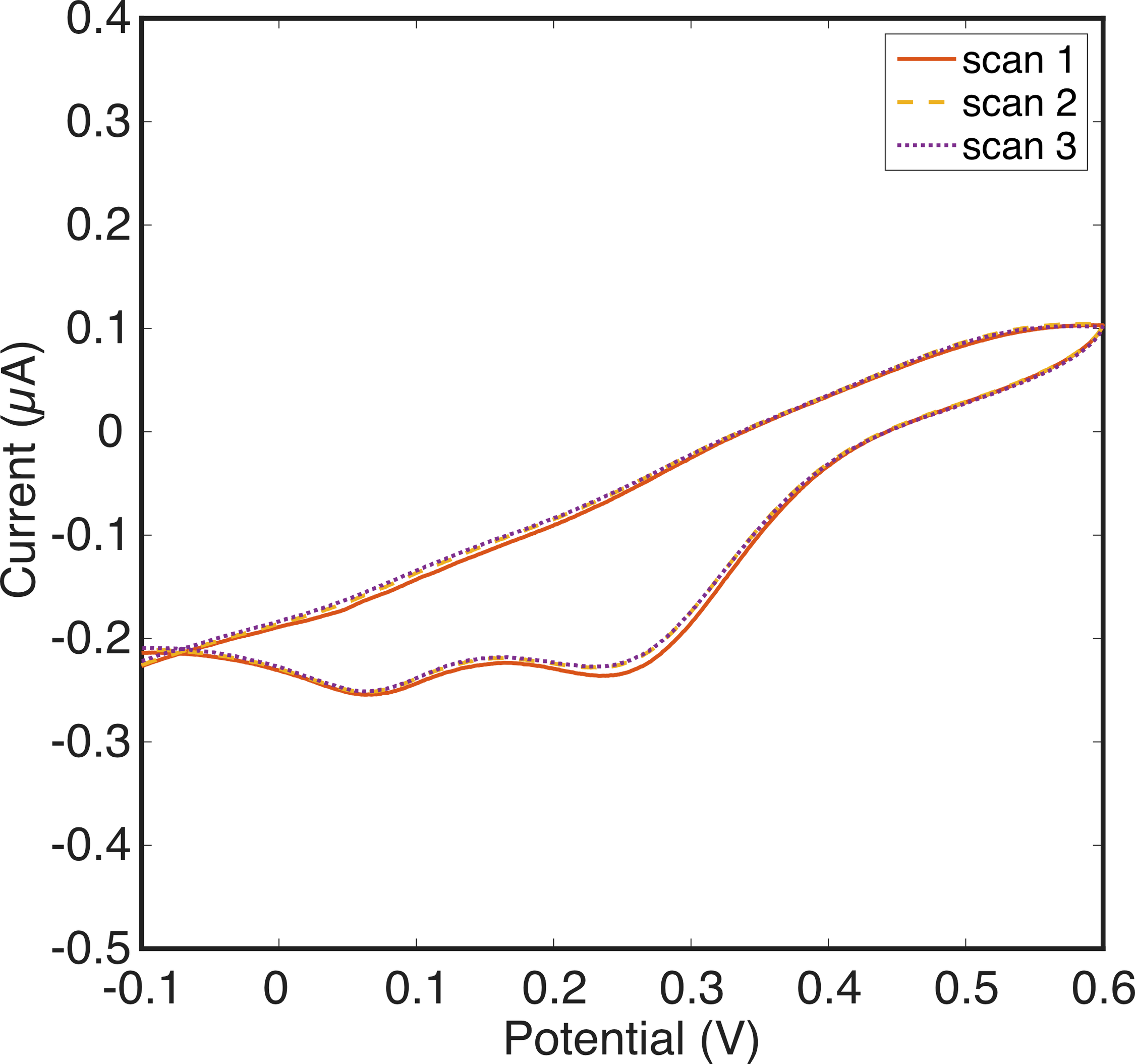

Supplement: S3 Fig — The consistency of the three measurements indicated that the electrode properties did not change. (TIFF) [file pone.0287824.s003.tiff]

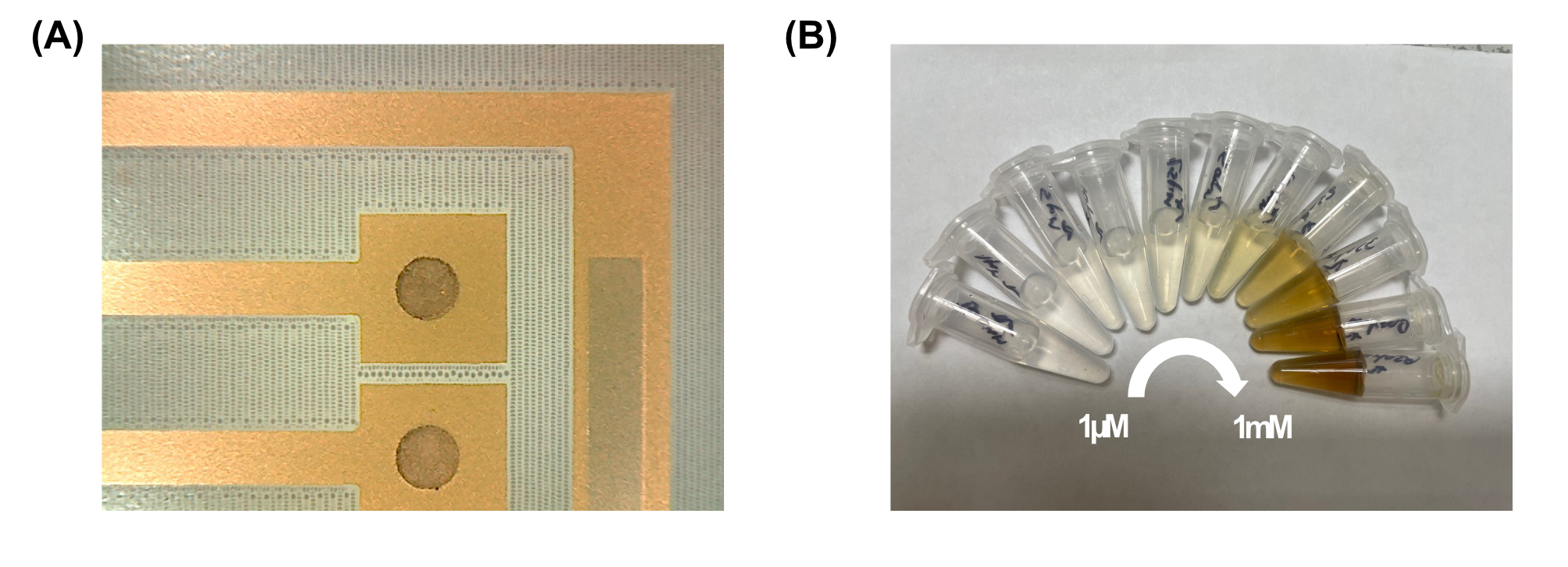

Supplement: S4 Fig — Pictures of (A) Au working electrodes after electrodeposition of AuNS, where newly formed Au layer can be observed physically; (B) A picture of solutions already containing 1mM FeCl3 upon addition of TC at a range of concentrations indicated on image, where colour changed with increasing TC concentration. (TIFF) [file pone.0287824.s004.tiff]

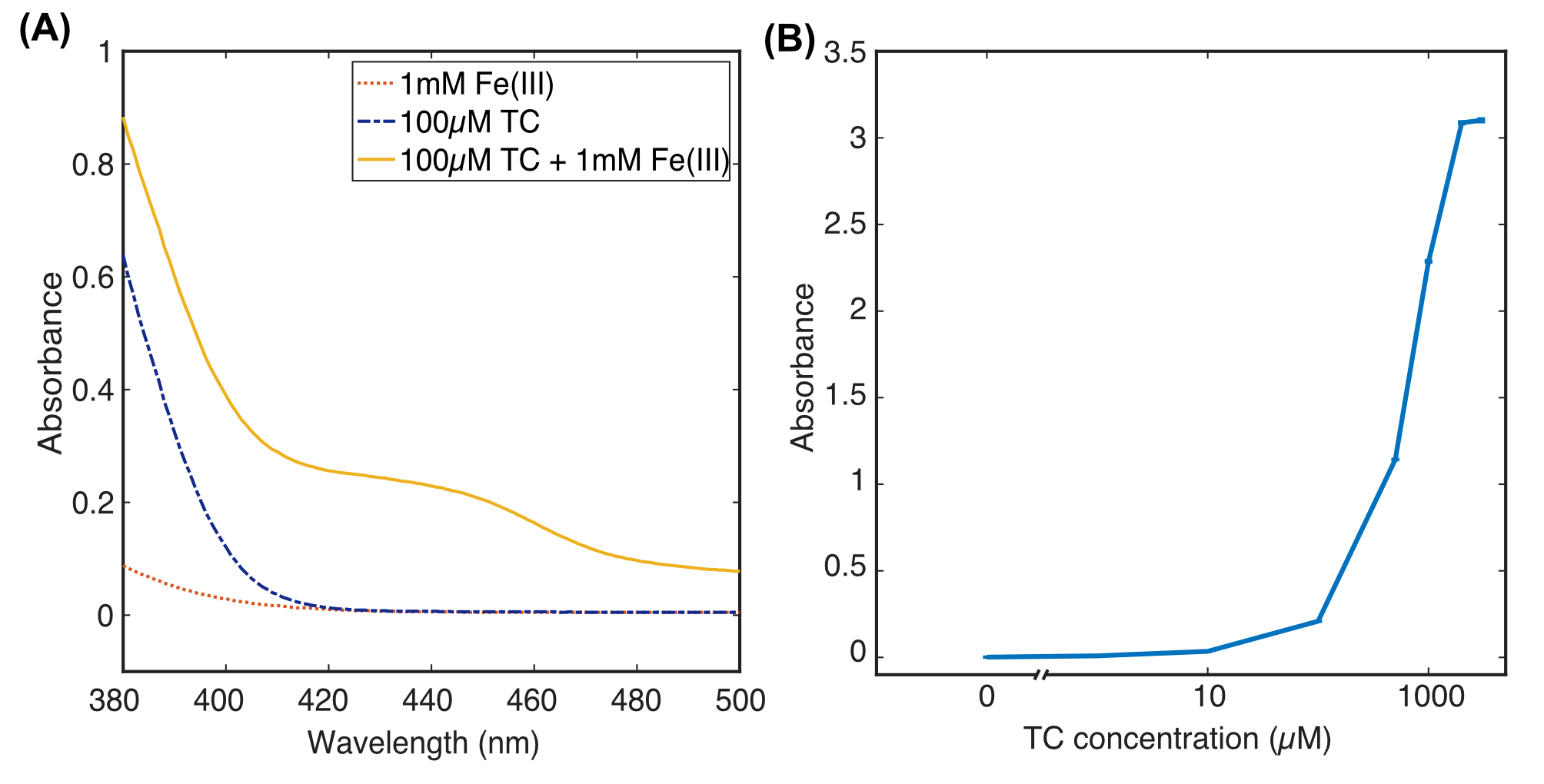

Supplement: S5 Fig — UV-Vis analysis of TC-Fe(III) solutions in pH 2.0; (A) UV-Vis spectrum of TC (100 μM) and FeCl3 (1 mM) separately and in a combination scanned between 380 and 500 nm, where a newly emerged peak appears at 444 nm only upon complex formation; (B) Proportional relationship between TC concentration range added to 1 mM Fe(III) vs. measured UV absorbance at 444 nm, resulting in a sigmoidal curve with saturation of the TC-Fe(III) complex at 2:1 ratio. (TIFF) [file pone.0287824.s005.tiff]

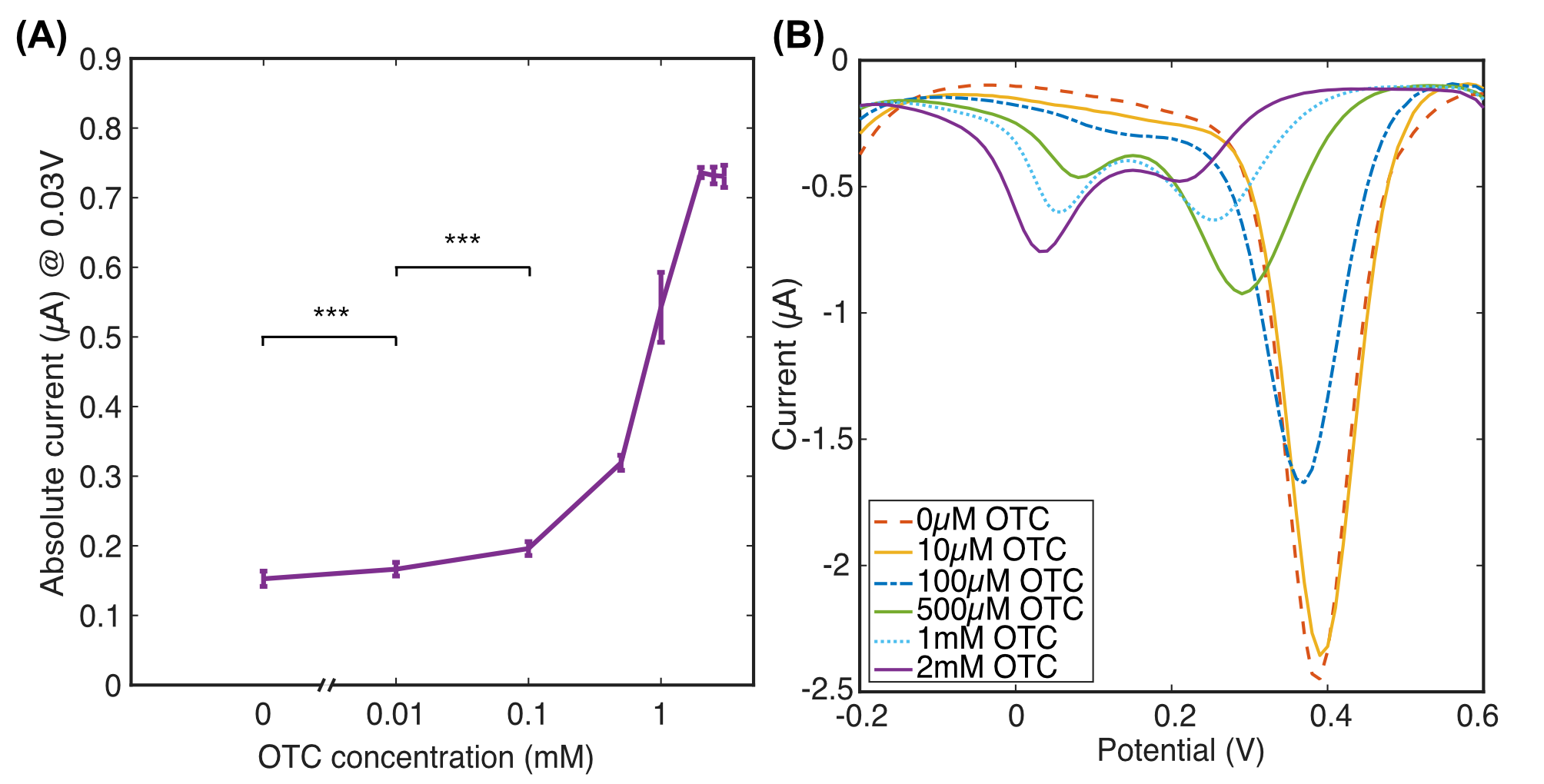

Supplement: S6 Fig — Electrode response to increasing OTC concentrations in the presence of 1 mM Fe(III), where (A) relationship between the measured absolute current (at 0.03 V) vs. OTC concentrations added to 1 mM Fe(III) resulting in a sigmoidal curve with error bars added that stand for the standard deviation between 8 replicate electrodes; t-test was performed, where obtained p-values were found as ***p < 0.001; (B) is DPV example of 1 mM Fe(III) when OTC is added at an increasing range of concentrations (indicated in the plot legend) scanned on plasma-treated Au/AuNS electrode. (TIFF) [file pone.0287824.s006.tiff]

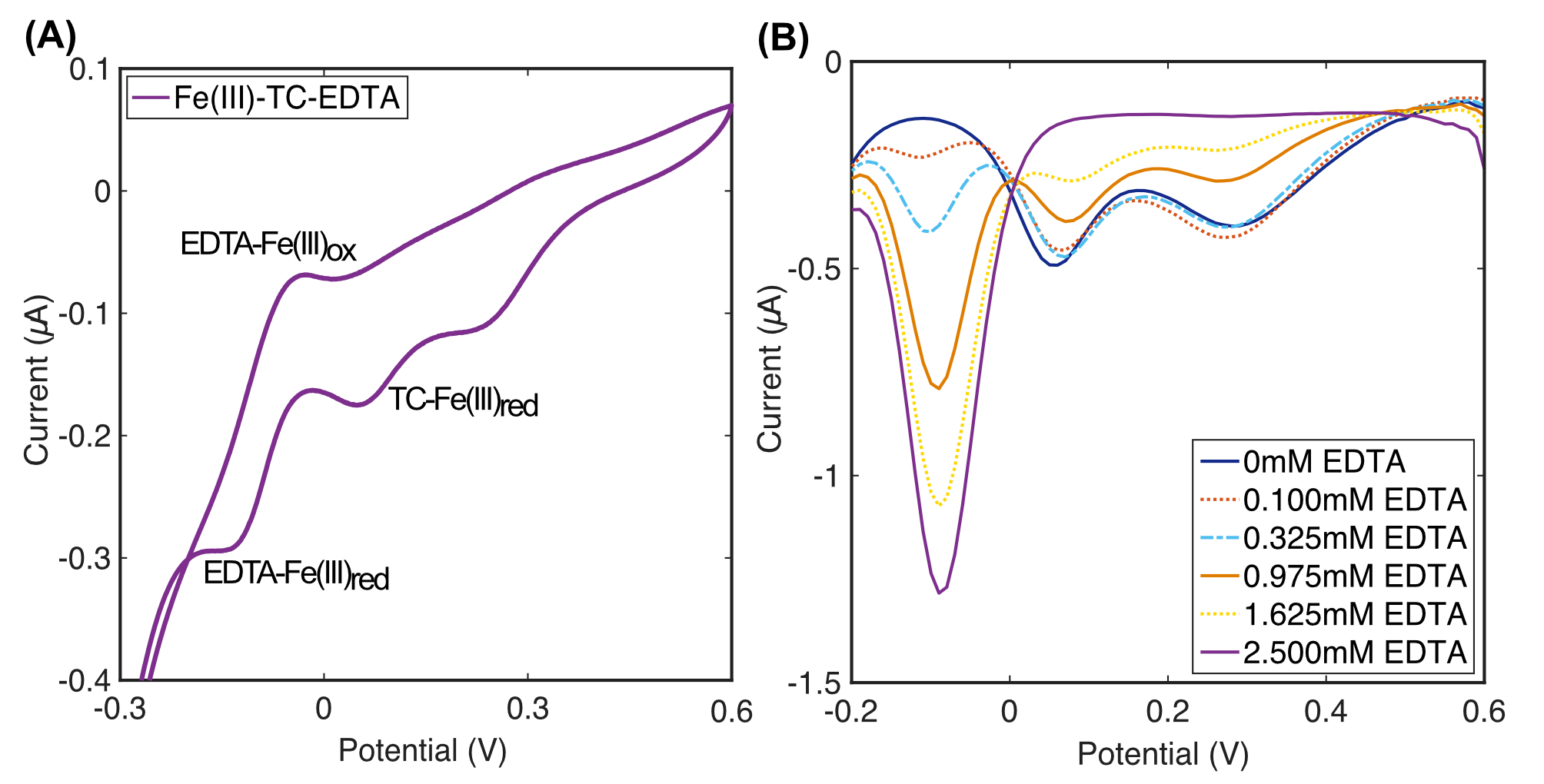

Supplement: S7 Fig — Stability of the TC-Fe(III) complex: EDTA complexes Fe(III) from TC and results in a fingerprint (scanned on Au/AuNS electrodes, n = 8) of the three compounds present simultaneously in a solution; (A) Upon addition of EDTA to TC-Fe(III) complex, a reversible redox couple was observed as oxidation peak at -0.07 V and reduction peak at -0.15 V; (B) The redox peak observed in DPV measurements was at -0.09 V (vs. Ag/AgCl QRE). This is consistent with the reported potential of the EDTA-Fe(III) redox couple by Allcorn et al. when considered in the context of the Ag/AgCl QRE used here and the scan rate of 50 mV/s [61]. The measured current of the peak consequently increases with further addition of EDTA which causes the TC-Fe(III) peak to decrease until it fully disappears from the DPV spectrum. This indicates that EDTA has pulled the TC-Fe(III) complexed iron apart, in addition to chelating all remaining free iron in the solution. Since TC is not electrochemically active on its own, only the peak that belongs to EDTA-Fe(III) remained in the scanned DPV spectrum. Based on these titration measurements, the amount of EDTA required to break the TC-Fe(III) and fully pull Fe(III) away into its own complex was found to be 2.5 mM or 1:2.5 ratio of TC:EDTA co-existing in a solution, respectively. (TIFF) [file pone.0287824.s007.tiff]

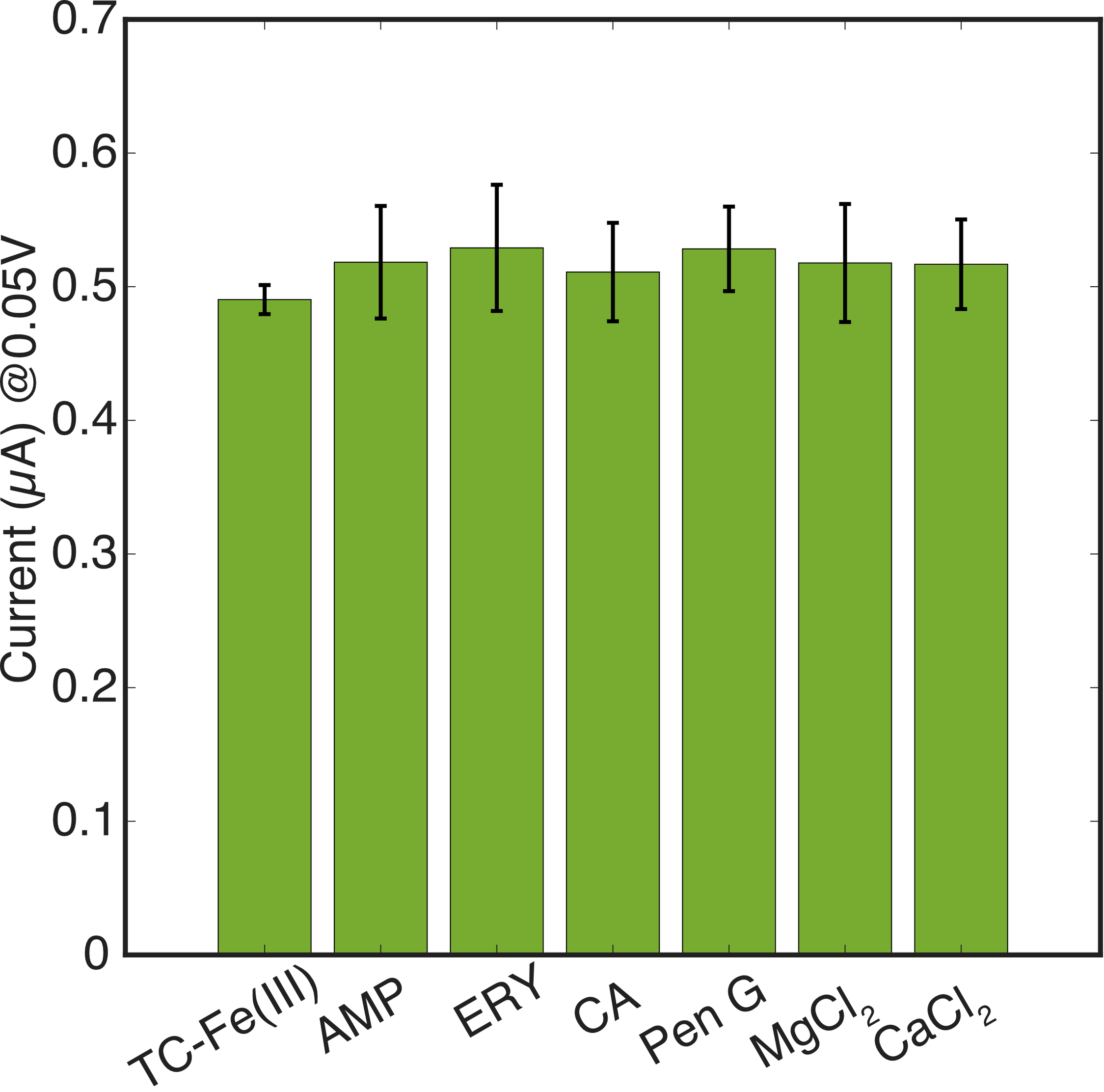

Supplement: S8 Fig — TC = tetracycline; AMP = ampicillin; CA = chloramphenicol; Pen G = penicillin G; ERY = erythromycin; MgCl2 = magnesium chloride; CaCl2 = calcium chloride. (TIFF) [file pone.0287824.s008.tiff]
